# IFN-λ drives distinct lung immune landscape changes and antiviral responses in human metapneumovirus infection

**DOI:** 10.1128/mbio.00550-24

**Published:** 2024-03-26

**Authors:** Jorna Sojati, Olivia B. Parks, Yu Zhang, Sara Walters, Jie Lan, Taylor Eddens, Dequan Lou, Li Fan, Kong Chen, Tim D. Oury, John V. Williams

**Affiliations:** 1 Department of Pediatrics, University of Pittsburgh School of Medicine, Pittsburgh, Pennsylvania, USA; 2 Department of Pulmonary, Allergy, and Critical Care Medicine, University of Pittsburgh School of Medicine, Pittsburgh, Pennsylvania, USA; 3 Department of Pathology, University of Pittsburgh School of Medicine, Pittsburgh, Pennsylvania, USA; 4 Department of Microbiology & Molecular Genetics, University of Pittsburgh School of Medicine, Pittsburgh, Pennsylvania, USA; 5 Institute for Infection, Immunity, and Inflammation in Children, UPMC Children’s Hospital of Pittsburgh, Pittsburgh, Pennsylvania, USA; Washington University in St. Louis, St. Louis, Missouri, USA

**Keywords:** interferon, human metapneumovirus, respiratory infection, host-pathogen immunity

## Abstract

Human metapneumovirus (HMPV) is a primary cause of acute respiratory infection, yet there are no approved vaccines or antiviral therapies for HMPV. Early host responses to HMPV are poorly characterized, and further understanding could identify important antiviral pathways. Type III interferon (IFN-λ) displays potent antiviral activity against respiratory viruses and is being investigated for therapeutic use. However, its role in HMPV infection remains largely unknown. Here, we show that IFN-λ is highly upregulated during HMPV infection *in vitro* in human and mouse airway epithelial cells and *in vivo* in mice. We found through several immunological and molecular assays that type II alveolar cells are the primary producers of IFN-λ. Using mouse models, we show that IFN-λ limits lung HMPV replication and restricts virus spread from upper to lower airways but does not contribute to clinical disease. Moreover, we show that IFN-λ signaling is predominantly mediated by CD45^-^ non-immune cells. Mice lacking IFN-λ signaling showed diminished loss of ciliated epithelial cells and decreased recruitment of lung macrophages in early HMPV infection along with higher inflammatory cytokine and interferon-stimulated gene expression, suggesting that IFN-λ may maintain immunomodulatory responses. Administration of IFN-λ for prophylaxis or post-infection treatment in mice reduced viral load without inflammation-driven weight loss or clinical disease. These data offer clinical promise for IFN-λ in HMPV treatment.

**IMPORTANCE:**

Human metapneumovirus (HMPV) is a common respiratory pathogen and often contributes to severe disease, particularly in children, immunocompromised people, and the elderly. There are currently no licensed HMPV antiviral treatments or vaccines. Here, we report novel roles of host factor IFN-λ in HMPV disease that highlight therapeutic potential. We show that IFN-λ promotes lung antiviral responses by restricting lung HMPV replication and spread from upper to lower airways but does so without inducing lung immunopathology. Our data uncover recruitment of lung macrophages, regulation of ciliated epithelial cells, and modulation of inflammatory cytokines and interferon-stimulated genes as likely contributors. Moreover, we found these roles to be distinct and non-redundant, as they are not observed with knockout of, or treatment with, type I IFN. These data elucidate unique antiviral functions of IFN-λ and suggest IFN-λ augmentation as a promising therapeutic for treating HMPV disease and promoting effective vaccine responses.

## INTRODUCTION

Acute respiratory infection (ARI) contributes to substantial mortality in children and adults worldwide and is the single largest cause of death in post-neonatal children. ARIs compromise 15% of all deaths in children under 5 years worldwide (1–3). Human metapneumovirus (HMPV), first described in 2001, causes upper and lower airway infection and is estimated to account for 4%–16% of ARI globally both in children and adults (4, 5). Although nearly all people are infected with HMPV during early childhood, immunity to HMPV is incomplete and re-infections occur often, with severe disease occurring among older adults and persons with underlying conditions. There are no approved antiviral therapies or vaccines against HMPV.

Type I and type III interferons (IFN) display strong antiviral activity and drive early innate responses to viral infections (6, 7). We showed previously that type I IFN contributes to HMPV immune-mediated pathogenesis and abrogation of type I IFN signaling reduced lung inflammation and lessened HMPV disease severity (8). Moreover, severe HMPV disease is not correlated with increased lung viral titer, suggesting pathogenesis may be driven by inflammation (9). However, the function of type III IFN (IFN-λ) in HMPV infection remains largely unknown. One existing study of IFN-λ in HMPV to date showed upregulation of both IFN-λ and type I IFN in A549 human lung carcinoma cells and bronchoalveolar lavage fluid (BAL) of BALB/c mice (10).

IFN-λ drives antiviral responses against pathogens that invade mucosal surfaces, particularly the respiratory and GI tracts (11, 12). IFN-λ is the dominant antiviral cytokine upregulated *in vivo* during infection with influenza A (13), respiratory syncytial virus (RSV) (14), and rotavirus (15) and induced at much higher levels than type I IFN in airway (influenza, RSV) and small intestine (rotavirus). Both respiratory and GI epithelial cells abundantly produce IFN-λ in several virus infection models (16–19), leading to the current model of epithelial cells as major producers of IFN-λ with other immune cells such as dendritic cells and macrophages as minor contributors (20–22). Favored production of IFN-λ by epithelial cells is thought to modulate a localized, less inflammatory antiviral response at barrier surfaces (23). IFN-λ exhibits potent activity against viruses that infect mucosal surfaces, including airways and GI tracts (24–28). Treatment with IFN-λ also restricts replication of influenza, RSV, SARS-CoV-2, rotavirus, and other viruses that invade barrier sites (29–36). Thus, IFN-λ is thought to play a major role in control of virus replication, making it a possible treatment modality worth pursuing. Studies of IFN-λ in influenza infection have uncovered key functional differences between responses to type I and IFN-λ, with IFN-λ treatment of influenza-infected mice protecting against disease while type I IFN treatment worsened disease (36, 37). These findings suggest that type I IFN, but not IFN-λ, contributes to pathogenesis during infection.

We sought to define the role of IFN-λ in human metapneumovirus pathogenesis and protection. We found that IFN-λ is essential for limiting lung HMPV replication and restricting virus spread from upper to lower airways but does not drive disease. Mice lacking IFN-λ signaling showed altered recruitment of lung myeloid and epithelial cells and inflammatory cytokine expression, suggesting a potential immunomodulation. Finally, treatment or prophylaxis with IFN-λ reduced viral burden without exacerbating disease, suggesting clinical promise for IFN-λ in HMPV treatment.

## MATERIALS AND METHODS

### Viruses

All HMPV isolates used were obtained from nasopharyngeal washes of patients with ARI as described previously (38). HMPV isolates TN/94-49 (subtype A2, low virulence) and C2-202 (subtype B1, high virulence) were grown in LLC-MK2 cells under enriched media conditions, sucrose-purified, and titered by plaque assay as described (39). Viral burden in mouse tissues was similarly titrated. Virus stocks used in animal studies had all undergone <10 passages in LLC-MK2 cells. HMPV viral stocks used for experiments underwent a maximum of three freeze/thaw cycles, which we have shown to not significantly change viral titer (40).

### Animals and cells

Six-to-eight-week-old C57BL/6J (B6) mice were purchased as either wild-type (WT) or *Ifnar1*^−/−^ (strain # 028288) from The Jackson Laboratory. *Ifnlr1*^−/−^ mice were generated using the University of Pittsburgh Innovation Technologies Development Core with the help of Dr. Sebastien Gingras. *Ifnlr1*^−/−^ mice were created using the CRISPR/Cas9 system to introduce a 262 base-pair deletion in exon 4 of the Ifnlr1 gene on chromosome 4 and generation of a premature stop codon 15 base-pairs into exon 5. B6 CD45.1 mice (strain #002014) were also purchased from The Jackson Laboratory for bone marrow transplant experiments. All animals were maintained in specific pathogen-free conditions in accordance with University of Pittsburgh Institutional Animal Care and Use Committee guidelines. Cell lines used for experiments included BEAS-2B non-transformed human bronchial epithelial cells (ATCC CRL-9609), A549 human lung carcinoma epithelial cells (ATCC CCL-185), LLC-MK2 monkey kidney cells (ATCC CCL-7), CMT64/61 mouse lung carcinoma epithelial cells (ECACC 86082105), and C10 non-transformed mouse type II alveolar epithelial cells (non-commercial; donated kindly by Dr. John Alcorn). Human and murine cell lines were authenticated by ATCC and ECACC short tandem repeat (STR) profiling prior to use. C10 cells were authenticated by flow cytometry staining for cell-specific alveolar type II epithelium markers prior to use.

### Infection and treatment models

For *in vivo* experiments, mice were anesthetized by inhaled isoflurane (5% isoflurane in 100% O_2_, flow rate 2.5 L/min) and infected intratracheally (I.T.) with 5 × 10^5^ plaque-forming units (PFU) of HMPV or mock LLC-MK2 cell lysate in 100 µL volume (38). For upper tract-limited infection, 5 × 10^5^ PFU HMPV was delivered intranasally (I.N.) to anesthetized mice in 10 µL total volume (5 µL per nostril). For treatment, recombinant human IFN-λ1 protein (Peprotech; catalog #300-02), recombinant mouse IFN-β protein (Sino Biologicals; catalog #50708), or recombinant mouse IFN-λ2 protein (Preprotech; catalog #250-33) were reconstituted in 0.1% bovine serum albumin (BSA). Anesthetized mice were given a 50 µL volume I.N. (25 µL per nostril) either 1 day prior to infection (prophylaxis) or 2 days post-infection (treatment). Mock prophylaxis or treatment involved same-day administration of an equal volume I.N. of 0.1% BSA alone. Mouse IFN-λ2 and IFN-β treatment were normalized for *in vivo* experiments to a similar degree of interferon-stimulated gene (ISG) induction, as described in Fig. S11; 1 μg of IFN-λ2 and 5 µg of IFN-β were administered.

For *in vitro* experiments, cells were inoculated with TN/94-49 or C2-202 HMPV. Multiplicity of infection (MOI), defined by the number of infectious viral particles per host cell, was optimized for similar degree of initial intracellular viral load with both HMPV strains, as described in Fig. S1. MOIs (PFU/cell) of 0.5 for TN/94-49 and 1 for C2-202 were used for CMT/64-61, MOIs of 0.1 for TN/94-49 and 1 for C2-202 were used for C10, MOIs of 0.1 for TN/94-49 and 0.5 for C2-202 were used for A549, and MOIs of 0.5 for TN/94-49 and 1 for C2-202 were used for BEAS-2B infection. Mouse IFN-λ2 treatment was optimized in *in vitro* experiments by the greatest degree of STAT1 phosphorylation (Fig. S1); 10 ng/mL IFN-λ2 were used for CMT/64-61 and C10 treatment. Human IFN-λ1 treatment was optimized in *in vitro* experiments by the greatest degree of ISG induction (Fig. S1); 100 ng/mL IFN-λ1 was used for A549 and 500 ng/mL IFN-λ1 was used for BEAS-2B treatment.

### Cytokine quantitation

Multiplex Luminex-based immunoassay (ProcartaPlex, Thermo-Fisher) or IL-28 Mouse ELISA (Thermo-Fisher; catalog #BMS6028) of undiluted lung homogenate was performed to determine inflammatory cytokine levels according to manufacturer’s instructions.

### qPCR

RNA was extracted from 100 µL volume of lung or nasal turbinate homogenate using RNeasy kit (Qiagen) according to manufacturer’s instructions. Quantitative reverse-transcription PCR (RT-qPCR) was performed in 25 µL reaction volume containing 5 µL extracted RNA using AgPath-ID One-Step RT-PCR (Applied Biosystems). TaqMan primers and probes were used according to manufacturer’s instructions (Applied Biosystems).

### Lung histopathology

A section of the lower left lung lobe was removed from euthanized mice and inflated to physiologic volume with 10% formalin using a 29.5-gauge syringe. Lung sections were embedded in paraffin, sectioned, stained with hematoxylin and eosin (H&E) by the UPMC Children’s Hospital of Pittsburgh Histology Core. Slides were imaged and scored at 200× magnification using a formal scoring system in a group-blinded fashion by a trained lung pathologist. Scoring criteria per field included: 0: no inflammation, 1:  <25% inflammation, 2: 25%–50% inflammation, 3: 50%–75% inflammation, 4:  >75% inflammation. To generate the histopathologic score, the score for each sample was added and divided by the total number of fields analyzed.

### Flow cytometry

We developed a protocol for simultaneous isolation of mouse lung myeloid and epithelial cells based on published studies (41–43). Following euthanasia, thoracotomy was performed to visualize the lungs and the left lung hilum was tied off using a suture to save for myeloid cell isolation. A tracheal incision was made, and the right lung was washed once with phosphate-buffered saline (PBS). For epithelial cell isolation, the right lung was inflated intratracheally with 0.5 mL dispase (Corning; 50 U/mL) immediately followed by 0.2 mL of 1% low melting point agarose and covered with ice for 5′ to create a solidified agarose plug. For both myeloid and epithelial cell preparations, lung tissue was digested enzymatically with 2 mg/mL collagenase A and 20 mg/mL DNase (Roche) for 60′ at 37°C and passed through a 70 µM cell strainer to obtain single-cell suspensions. Cells were then incubated with ACK lysis buffer (Sigma-Aldrich). Cells were stained with either myeloid or epithelial cell markers (Table S1) using the following protocol: LIVE-DEAD violet dye (Thermo-Fisher) for 20′ at room temperature (RT), surface antibodies for 45′ at 4°C (1 µL antibody/sample in BD Horizon Brilliant Stain buffer; catalog #566349), fixation with FOXP3 fix/permeabilization buffer for 20′ at 4°C (Invitrogen; catalog #50-112-8857), intracellular markers for 30′ at 4°C (1 µL antibody/sample in FOXP3 permeabilization buffer), and washes with FACS buffer (1% fetal bovine serum in PBS). Cells were resuspended in 300 µL in FACS buffer + 100 µL Counting Beads (Biolegend), strained through nylon 70 µM filter and analyzed on an Aurora spectral cytometer (Cytek Biosciences). Unstained cells from each experiment were collected, fixed with 4% PFA, and used for spectral unmixing to subtract cellular autofluorescence. Data were analyzed using FlowJo software (Tree Star). Fluorescence minus one controls are used for gating of macrophage, dendritic cell, and epithelial cell subsets. Fluorescence-activated cell sorting (FACS) experiments were performed using a FACSAria III Cell Sorter (BD Biosciences).

### IFN-λ staining

For IFN-λ staining of cells, C10 or CMT/64-61 cells infected with HMPV at multiplicity of infection (MOI) of 1 were treated 18 h post-infection with brefeldin A (BD Biosciences) at 1:200 dilution. Cells were collected 6 h later via gentle dissociation using Tryp-LE (Thermo-Fisher) and stained with LIVE/DEAD violet dye (Thermo-Fisher), epithelial panel markers (Table S1), and anti-IL-28 mAb (clone D12, Santa Cruz Biotechnology). For IFN-λ staining of mouse lung cells by flow cytometry, HMPV-infected mice were injected 18 h post-infection intraperitoneally (I.P.) with 250 µg brefeldin A (Cayman Chemicals; catalog #11861) reconstituted in dimethyl sulfoxide (DMSO). At 24 h post-infection, lung tissue was isolated and processed into single-cell suspensions as above, and cells were stained with LIVE/DEAD violet dye, myeloid and epithelial panel markers (Table S1), and anti-IL-28 at 1:500 (optimized by antibody titration). For IFN-λ staining of mouse lung cells by PrimeFlow assay (Thermo-Fisher), tissue was harvested from HMPV-infected mice 24 h post-infection and processed into single cell suspensions as above and and stained with LIVE/DEAD violet dye, myeloid and epithelial cell markers (Table S1), and an *Ifnl3* target-specific probe set with Alexa Fluor 647 label (Thermo-Fisher). The PrimeFlow assay was performed per the manufacturer’s instructions (Thermo-Fisher). Fluorescence-minus-one control and isotype control (irrelevant T-cell marker RORγT conjugated to the same fluorophore as IL-28 mAb) were used for gating and appropriate subtraction of background staining.

### Bone marrow transplant

Recipient *Ifnar1*^−/−^ and *Ifnlr1*^−/−^ mice were administered 10 centigray (cGy) total body irradiation 1 day prior to transplant using a MultiRad 350 X-Ray irradiation system (Precision X-Ray). Irradiation was given in two split doses delivered 4 h apart. The next day, donor bone marrow was collected from *Ifnar1*^−/−^, *Ifnlr1*^−/−^, or CD45.1 mice. Femurs and tibias of donor mice were harvested and flushed twice with 10 mL D10 media (10% FBS, 1% penicillin-streptomycin, 1% L-glutamine, 1% MEM non-essential amino acids, 0.1% 50 mM β-mercaptoethanol) using an 18G needle. Cells were spun down at 1300 RPM for 5 min, passed through a 70-µM cell strainer to obtain single-cell suspensions, and resuspended in sterile PBS. 1 × 10^7^ bone marrow cells in a 200 µL suspension were injected into irradiated mice by tail vein injection. Mice were given sterile autoclaved water and irradiated food and left to rest for 6 weeks post-transplantation before being infected.

### Single-Cell RNA sequencing

To ensure representation of both epithelial and hematopoietic lineage cells for each mouse sample, both right and left lungs were inflated with a lower volume (0.25 mL) dispase with agarose plug followed by digestion and processing into single-cell suspension as described above. Cells were next washed 5 times with 5–10 mL of PBS + 10% FBS and centrifuged at 500 × *g* for 5′, followed by a final wash and centrifugation at 200 × *g* for 10′ to remove debris. Dead cells were removed by magnetic separation using Annexin V kit (STEMCELL Technologies). Using the 10× Genomics 3′ CellPlex Kit, cells from each mouse sample were tagged using lipid-conjugated Barcode oligonucleotides according to manufacturer’s instructions. Cells were passed through a 40-µM filter and cell viability was determined using Cellometer 2000 (all showed >90% viability) prior to loading on 10× Chromium instrument. Following established techniques using Chromium Single Cell 3′ Library V2 kit (10×), libraries were constructed and RNA-seq was performed on each sample targeting 20,000 reads per cell. Sequencing outputs were processed with Cellranger.

### Statistical analyses

All experiments were conducted in triplicate and repeated at least twice.

scRNA-seq was performed in duplicate, and data were analyzed using Seurat. Individual samples were demultiplexed by Barcode oligonucleotides. Doublets identified by HTOs and poor-quality droplets (if deficient number of genes detected) were excluded from analysis. Gene expression was assessed using Wilcoxon rank-sum with Bonferroni correction. All other data analysis was performed using Prism version 9.0 (GraphPad software). Individual comparisons were done for all figures using student’s *t*-test. Multiple group comparisons were done for all figures using either one-way (if one independent variable) or two-way (if two or more independent variables) ANOVA. Data are represented as mean + standard deviation. *P* values less than 0.05 were considered statistically significant. The statistical test used and *P* values are indicated in each figure legend.

## RESULTS

### IFN-λ is upregulated during HMPV infection and reduces titer in epithelial cells

**Fig 1:**
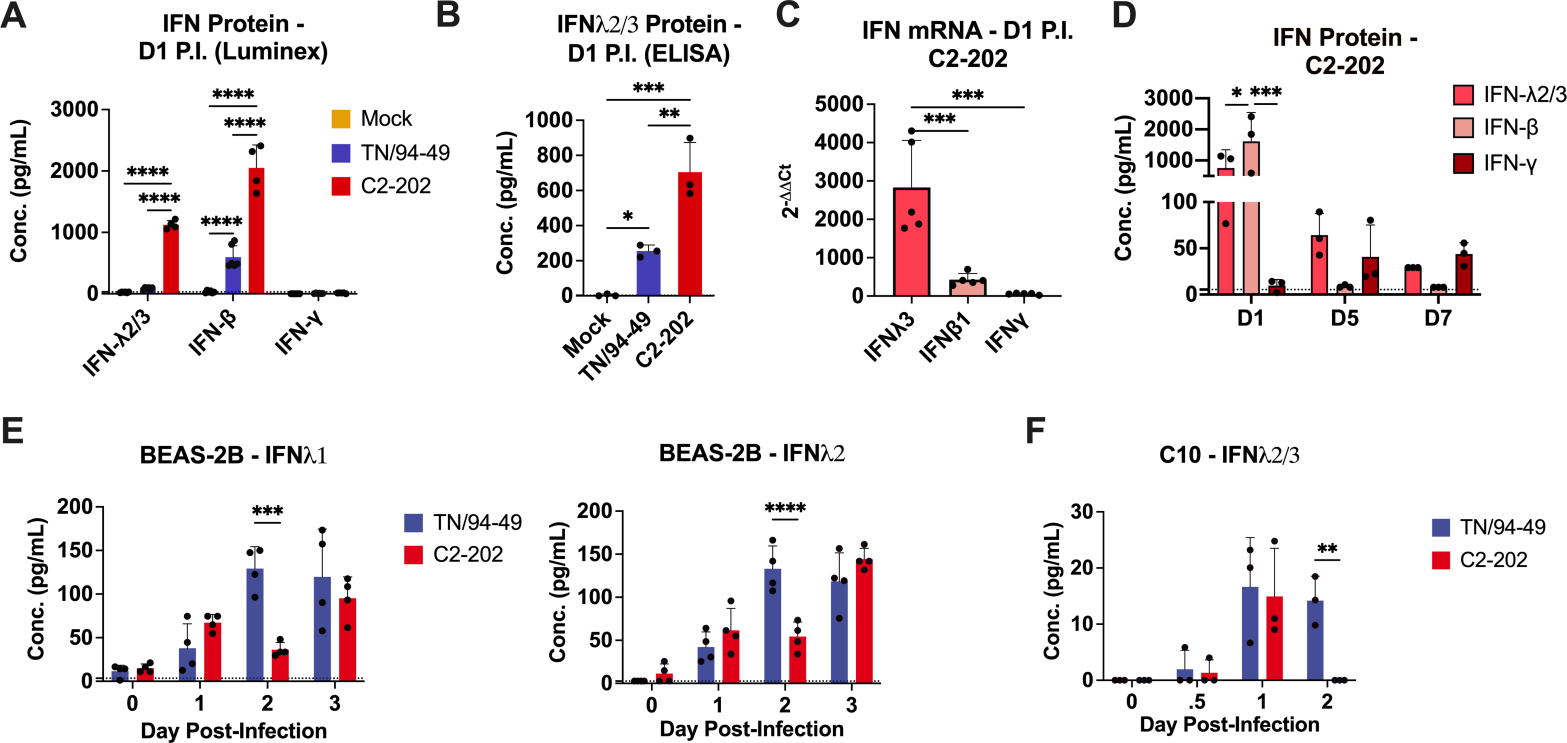
IFN-λ is highly upregulated during HMPV infection. Mice were infected with 5 × 10^5^ PFU of HMPV strains TN/94-49 or C2-202 or mock cell lysate and IFN-λ2/3, IFN-β, and IFN-γ were measured in lung homogenate day 1 post-infection by (A) Luminex; (**B**) ELISA; or (C) qPCR. Data in C were normalized to HPRT1 gene and mock-infected mice by the 2^−∆∆*Ct*^ method. (**D**) Mice were infected with HMPV C2-202 and IFN-λ2/3, IFN-β and IFN-γ were measured in lung homogenate days 1, 5, and 7 post-infection by Luminex. (**E**) BEAS-2B cells were infected with TN/94-49 or C2-202 and IFN-λ1 and IFN-λ2 measured in supernatant days 0–3 post-infection by Luminex. (**F**) C10 cells were infected with TN/94-49 or C2-202 and IFN-λ1 and IFN-λ2 measured in supernatant days 0–3 post-infection by Luminex. Cells were infected at multiplicity of infection (MOI) of 0.05. Limit of detection noted by dashed line. Data are shown as mean ± standard deviation. Analyses by student’s one-way or two-way ANOVA. *P < 0.05, **P < 0.01, ***P < 0.001, ****P < 0.0001.

We first determined whether IFN-λ was upregulated by HMPV using several models of *in vitro* and *in vivo* infection. We infected mice with the low-virulence TN/94-49 HMPV strain (44) and observed upregulation of type I IFN (IFN-β) and IFN-λ, but not type II IFN (IFN-γ), in mouse lung at an early time point (day 1) post-infection (Fig. 1A and B). IFN-λ quantification in mouse lung by both Luminex and ELISA showed increased expression with C2-202, a more virulent strain of HMPV (44) (Fig. 1A and B). IFN transcript levels in mouse lung by qPCR also showed upregulation of IFN-λ and IFN-β on day 1 post-infection although IFN-λ to a higher degree (Fig. 1C). This did not correlate to IFN protein levels, which were slightly higher for IFN-β in C2-202-infected mice (Fig. 1A). We measured kinetics of IFN-β and IFN-λ over time and found IFN-β was increased day 1 post-infection but decreased to undetectable levels in mouse lung by day 5 (Fig. 1D). IFN-λ was also highly upregulated on day 1 and remained at detectable levels on days 5 and 7 post-infection.

As a complementary approach, we assessed whether IFN-λ was upregulated by HMPV infection of immortalized, non-tumorigenic human and mouse lung epithelial cell lines. IFN-λ1 and IFN-λ2 were increased by both HMPV strains in BEAS-2B human bronchial cells, with expression increasing over time (Fig. 1E). Similarly, IFN-λ2/3 was increased in C10 mouse alveolar type II cells, peaking on day 1 post-infection (Fig. 1F). Collectively, these data show that low and high virulence HMPV strains induce IFN-λ *in vivo* in mouse lung and in both human and murine epithelial cell *in vitro* systems.

We next sought to test whether IFN-λ exerts antiviral effects in mouse or human lung epithelial cells. First, we optimized IFN-λ dose by measuring induction of downstream effects, either STAT1 phosphorylation in mouse cell lines (Fig. S1A and B) or expression of interferon-stimulated genes MX1 and IRF9 for human cell lines (Fig. S1D). For all cell lines, we also optimized viral inoculation dose to use MOIs that led to comparable degrees of initial intracellular HMPV load of HMPV strains TN/94-49 or C2-202 (Fig. S1C and E). We then infected either untreated cells or cells pre-treated with IFN-λ 24 h prior with HMPV and assessed viral burden over time (12, 24, or 48 h post-infection) by intracellular titer. All cell lines tested showed lower intracellular HMPV titers over time with IFN-λ treatment (Fig. S2). The antiviral effect of IFN-λ reduced titer of both TN/94-49 and C2-202. IFN-λ treatment also led to lower HMPV supernatant titers in all cell lines (Fig. S3). Taken together, we uncovered antiviral effects of IFN-λ in four *in vitro* HMPV infection models.

### Type II alveolar cells are primary IFN-λ inducers in HMPV infection

**Fig 2:**
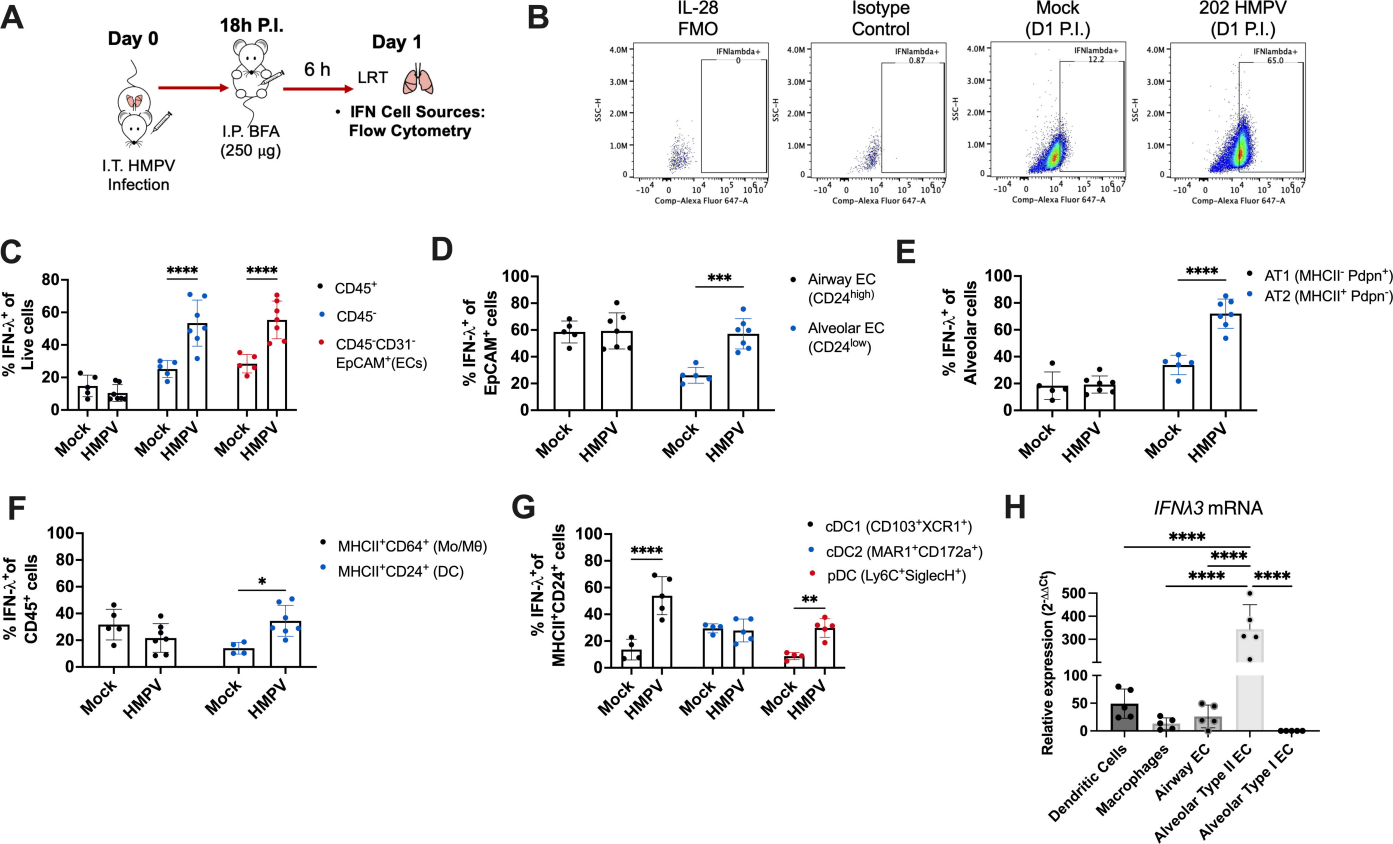
Alveolar type II epithelial cells are primary IFN-λ inducers during early HMPV infection. Mice were infected with 5 × 10^5^ PFU of HMPV strain C2-202 or mock cell lysate and IFN-λ expression in mouse lungs was measured by flow cytometry day 1 post-infection. (**A**) Schematic diagram of flow cytometric IFN-λ staining. (**B**) Gating for IFN-λ expression on type II alveolar epithelial cells. Fluorescence-minus-one control and isotype control (irrelevant T-cell marker RORγT conjugated to the same fluorophore) were used. For C–G, IFN-λ expression was assessed in lung epithelial and myeloid populations of mock- or C2-202 HMPV-infected mice day 1 post-infection. (**C**) Frequency of IFN-λ2/3^+^ cells in CD45^+^ (immune) vs CD45^−^ (non-immune) populations. (**D**) Frequency of IFN-λ2/3^+^ cells in alveolar vs airway epithelial cell types. (**E**) Frequency of IFN-λ2/3^+^cells in alveolar cell subpopulations in lungs of mice, showing upregulation of IFN-λ in type II alveolar epithelial cells with HMPV infection. (**F**) Frequency of IFN-λ2/3^+^ cells in parent populations of macrophages vs dendritic cells. (**G**) Frequency of IFN-λ2/3^+^ cells in dendritic cell subpopulations. Data are shown as mean ± standard deviation. Analyses were done by two-way ANOVA. *P < 0.05, **P < 0.01, ***P < 0.001, ****P < 0.0001.

We next sought to determine which cells were the primary producers of IFN-λ in HMPV infection. We used two flow cytometric panels based on prior studies (41, 45–47) to identify (1) mouse lung myeloid cells, including monocyte, macrophage, and dendritic cell subsets and (2) mouse lung epithelial cells, including differentiation of alveolar and airway epithelial cell subsets (Fig. S4). All *in vivo* studies used C2-202 since this virulent HMPV strain induced higher IFN-λ (Fig. 1A and B). We validated a commercial fluorophore-conjugated mAb to mouse IFN-λ (IL-28) *in vitro* using mouse lung epithelial cells (Fig. S5A). As a negative control, we confirmed by ELISA that HMPV-infected C10, but not CMT/64-61, cells produced IFN-λ (Fig. S5B). Intracellular staining using the mouse IFN-λ mAb detected IFN-λ expression in HMPV-infected C10, but not CMT/64-61 cells (Fig. S5C). Next, we used the mAb to measure IFN-λ in mouse lung by flow on day 1 post-infection after mice were infected with C2-202 and treated with brefeldin to enhance intracellular accumulation of secreted proteins (Fig. 2A). IFN-λ expressing cells were increased with HMPV infection, particularly in CD45^−^ populations and epithelial cells (Fig. 2B and C). Airway epithelial cells and especially alveolar type II cells showed high IFN-λ production with HMPV infection (Fig. 2D and E; Fig. S6A and B). We also found that dendritic cells, particularly conventional type I dendritic cells (cDC1) and plasmacytoid dendritic cells (pDC), upregulated IFN-λ (Fig. 2F and G; Fig. S6C). IFN-λ expression in alveolar type II cells decreased over days 3 and 5 post-infection (Fig. S6D and E), paralleling our previously observed IFN-λ kinetics (Fig. 1D). These findings demonstrate that alveolar type II cells are major producers of IFN-λ in early HMPV infection, with cDC1 and pDC also minor producers of IFN-λ.

These results were further supported by qPCR of FACS-sorted myeloid and epithelial cell populations from lungs of mice infected with C2-202 and harvested 1 day post-infection, showing the highest *Ifnl3* mRNA expression in alveolar type II cells (Fig. 2H). We also used the complementary approach of a PrimeFlow RNA assay to couple flow cytometry with amplification and detection of a target-specific *Ifnl3* mRNA probe (Fig. S7). These data also show CD45^−^ populations, specifically epithelial cells and in particular alveolar type II cells, with the highest *Ifnl3* expression in lungs of C2-202 infected-mice day 1 post-infection (Fig. S7).

**Fig 3:**
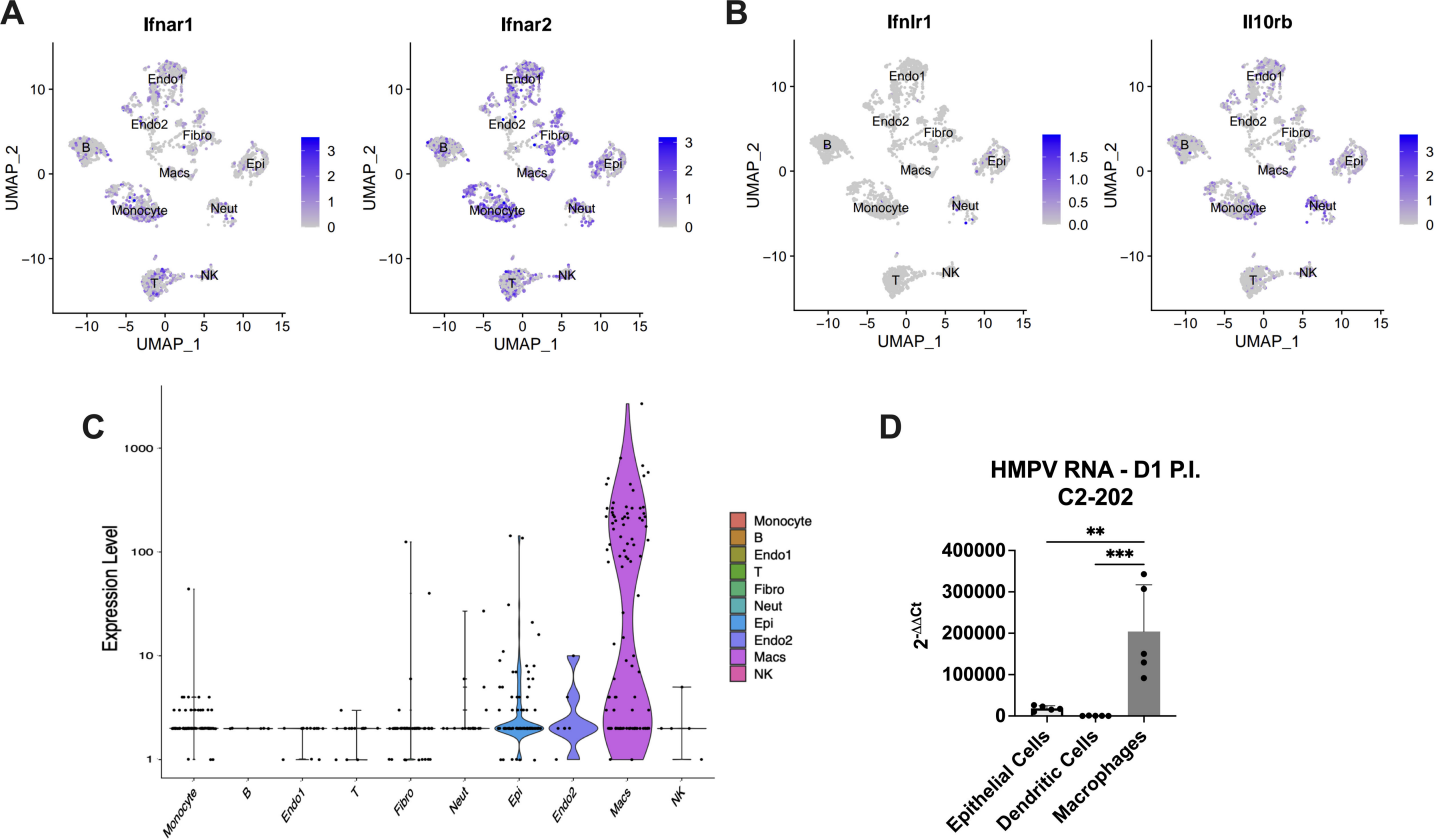
Epithelial cells respond to IFN-λ and internalize HMPV in early infection. Single-cell RNA sequencing (scRNA-seq) was performed on cells isolated from lungs of mice infected with 5 × 10^5^ PFU C2-202 HMPV and harvested day 1 post-infection. (**A**) Type I IFN receptor expression was measured. Gene expression of subunits *Ifnar1* (right) and *Ifnar2* (left) stratified by lung cell type. (**B**) IFN-λ receptor expression was measured. Gene expression of subunits *Ifnlr1* (right) and *IL10Rb* (left) stratified by lung cell type. (**C**) Expression levels of HMPV transcripts was analyzed and stratified by cell type. (**D**) qPCR of HMPV expression was done for FACS-sorted populations from lungs of C2-202-infected mice harvested day 1 post-infection. Data are normalized to the HPRT1 gene and the null condition of mock-infected mouse lung homogenate by the 2^−∆∆*Ct*^ method. Analysis was done by one-way ANOVA. **P < 0.01, ***P < 0.001.

We next explored whether cell specificity of IFN-λ induction correlated to receptor expression. Using single-cell RNA-sequencing (scRNA-seq), we measured the expression of either type I (*Ifnar1* and *Ifnar2*) or type III (*Ifnlr1* and *Il10rb*) IFN receptor subunits in mouse lung cells on day 1 post-infection. Both type I IFN receptor subunits were expressed in various cell types (Fig. 3A); in contrast, while *Il10rb* was expressed by many cell types, *Ifnlr1* was only expressed on epithelial cells and neutrophils (Fig. 3B). We also tested whether there was correlation with HMPV infection by analyzing HMPV-specific RNA in the scRNA-seq data set. We found that the highest detection of HMPV RNA was in macrophages, with epithelial and endothelial cells also showing low expression (Fig. 3C). This was confirmed by qPCR of FACS-sorted cells, showing the highest HMPV RNA in macrophages and lower expression in epithelial cells (Fig. 3D). These suggest that in early HMPV infection, when IFN-λ is highest, the most internalized virus is likely from phagocytosis rather than active replication. We next assessed whether there was correlation to induction of interferon-stimulated genes (ISGs) using this scRNA-seq data set. We found that several ISGs associated with type I and type III IFN signaling were upregulated with HMPV infection (Fig. S8A), but that upregulation for all was broad across myeloid, epithelial, and stromal cell populations rather than specific to HMPV-infected cells or cells producing IFN-λ (Fig. S8B). Thus, epithelial cells are primary producers of IFN-λ during infection, specifically express type III IFN receptor, and respond to HMPV in both infected epithelial cells and macrophages that phagocytose virus.

### IFN-λ controls HMPV replication without contributing to disease primarily through CD45^−^ non-immune cell-mediated mechanisms

**Fig 4:**
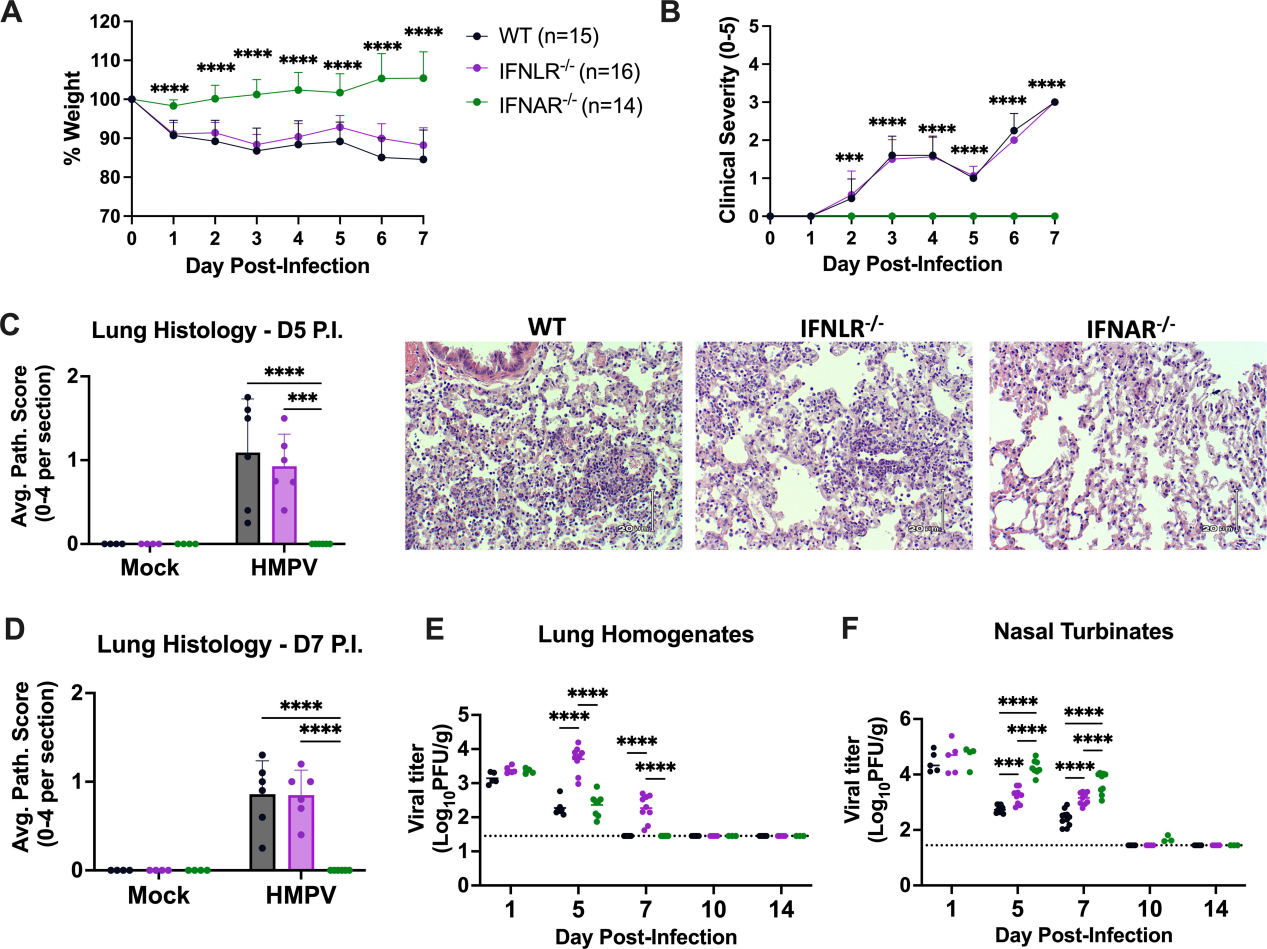
IFN-λ limits HMPV lung replication without contributing to inflammatory disease. C57BL/6 (**B6**) mice (shown in black), *Ifnar1^−/−^* (called IFNAR^−/−^, shown in green), and *Ifnlr1^−/−^* (called IFNLR^−/−^, shown in purple) were infected with 5 × 10^5^ PFU C2-202 HMPV and disease assessed by measuring body weight (**A**) and clinical severity scores (**B**) to day 7 post-infection. Weight represented as % of day 0. Clinical severity scores were measured by assigning 1 point out of 5 for each of the following: hunching, huddling, fur ruffling, rapid breathing, and lethargy. Analyses were done by two-way ANOVA. ***P < 0.001, ****P < 0.0001 for IFNAR^−/−^ vs both WT and IFNLR^−/−^. (**C**) Lung histology was performed for 5 × 10^5^ C2-202 HMPV-infected mice euthanized day 5 or day 7 post-infection. Scoring criteria per field included: 0: no inflammation; 1: <25% inflammation; 2: 25%–50% inflammation; 3: 50%–75% inflammation; 4: >75% inflammation. Score for each sample was added and divided by total number of fields analyzed. Representative lung histology images shown on right. (**C, D**) HMPV titer (PFU/g) was measured in lung homogenates (**E**) or nasal turbinates (**F**) of 5 × 10^5^ C2-202 HMPV-infected mice day 5 post-infection. Limit of detection noted by dashed line. Analyses were done by two-way ANOVA, ***P < 0.001, ****P < 0.0001.

To explore functions of type I IFN vs IFN-λ, we infected wild-type (WT) mice, mice lacking type I IFN receptor (*Ifnar1^−/^*^−^), or mice lacking type III IFN receptor (*Ifnlr1^−/^*^−^). Mice lacking type I IFN signaling alone showed no weight loss, clinical disease, or lung pathology, while *Ifnlr1*^−/−^ mice showed similar degrees of weight loss, clinical disease, and lung inflammation as WT mice (Fig. 4A through D). These suggest that unlike type I IFN, IFN-λ signaling does not contribute to HMPV disease. We also assessed HMPV burden over time. *Ifnlr1*^−/−^ mice exhibited higher lung HMPV titers than WT or *Ifnar1^−/−^* mice by day 5 and day 7 post-infection and delayed viral clearance, showing that IFN-λ contributes to limiting HMPV replication (Fig. 4E). This appeared to be airway site-specific, as both *Ifnar1*^−/−^ and *Ifnlr1*^−/−^ mice showed higher nasal turbinate titers compared to wild-type mice (Fig. 4F). Thus, IFN-λ specifically limits lung HMPV replication without contributing to inflammatory pathogenesis.

Given the distinct phenotypes of disease contribution by type I IFN and control of HMPV lung replication by IFN-λ, we sought to determine if these responses were mediated by immune or non-immune cell contributors using bone marrow transplants. *Ifnar1*^−/−^ and *Ifnlr1*^−/−^ recipient mice were irradiated and given donor bone marrow from either wild-type B6 (CD45.1) mice or donor *Ifnar1*^−/−^ or *Ifnlr1*^−/−^ as controls (Fig. S9A). Here, we saw that the phenotype of disease rescue in *Ifnar1*^−/−^ was maintained in control mice but lost in mice receiving wild-type (CD45.1) bone marrow (Fig. S9B and C). However, control of HMPV lung replication was not restored in *Ifnlr1*^−/−^ mice receiving wild-type CD45^+^ cells, as there were similarly high lung HMPV titers in *Ifnlr1*^−/−^ control mice and *Ifnlr1*^−/−^ mice receiving CD45.1 bone marrow (Fig. S9D). Therefore, while type I IFN responses were largely mediated by CD45^+^ immune cells, IFN-λ responses appear to be primarily mediated by CD45^−^ non-immune cell-mediated mechanisms.

### IFN-λ alters recruitment of lung macrophages and loss of ciliated epithelial cells

**Fig 5:**
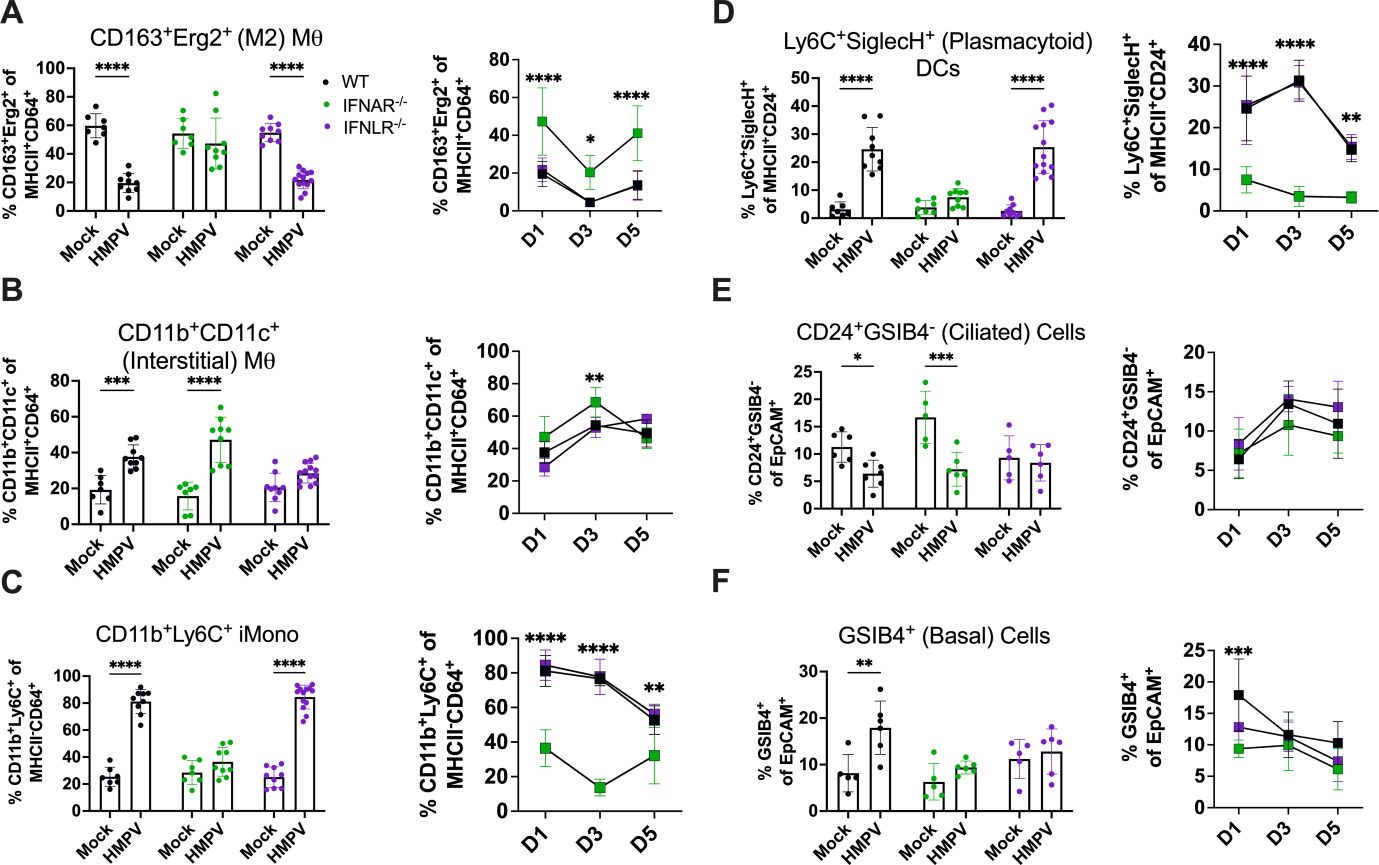
IFN-λ promotes differential lung innate cell recruitment. WT (shown in black), *Ifnar1*^−/−^ (labeled IFNAR^−/−^, shown in green), and *Ifnlr1*^−/−^ (labeled IFNLR^−/−^, shown in purple) mice were infected with 5 × 10^5^ PFU C2-202 HMPV, and lung myeloid and epithelial cell populations quantified day 1 post-infection by flow cytometry. (**A–D**) (Left) Frequency of lung innate immune cell populations, including M2 macrophages (**A**), CD11b^+^CD11c^+^ interstitial macrophages (**B**), inflammatory monocytes (**C**), and plasmacytoid dendritic cells (**D**) day 1 post-infection. (Right) Frequency of lung immune cell populations of C2-202 HMPV-infected mice from days 1–5 post-infection. (**E, F**) (Left) Frequency of lung epithelial cell populations, including ciliated (**E**) or basal (**F**) epithelial cells day 1 post-infection. (Right) Frequency of lung epithelial cell populations of C2-202 HMPV-infected mice from days 1–5 post-infection. Data are shown as mean ± standard deviation. Analyses were done by two-way ANOVA. *P < 0.05, **P < 0.01, ***P < 0.001, ****P < 0.0001.

Given the different functions of type I IFN vs IFN-λ in HMPV infection, we next tested whether this correlated to differences in lung innate immune cell recruitment by flow cytometry of lung cells in mock- vs HMPV-infected WT, *Ifnar1^−/−^*, and *Ifnlr1^−/−^* mice early post-infection. CD163^+^Erg2^+^ M2 macrophages were reduced on day 1 post-infection in WT and *Ifnlr1*^−/−^ mice but present in *Ifnar1*^−/−^ mice, suggesting type I IFN drives depletion of M2 macrophages early post-infection (Fig. 5A). Intact IFN-λ signaling may also be important to maintain M2 macrophages. CD11b^+^CD11c^+^ interstitial macrophages were increased with HMPV infection in all mice except *Ifnlr1*^−/−^, suggesting IFN-λ is important for their recruitment (Fig. 5B). All mice except *Ifnar1*^−/−^ showed increased CD11b^+^Ly6C^+^ inflammatory monocytes and increased Ly6C^+^SiglecH^+^ pDC, suggesting type I IFN is important for their recruitment (Fig. 5C and D). *Ifnar1*^−/−^ mice, which showed rescue of clinical disease, maintained high M2 macrophages, low inflammatory monocytes, and low pDC out to day 3 and day 5 post-infection, implicating these populations in HMPV pathogenesis (Fig. 5A, C and D). All mice except *Ifnlr1*^−/−^ mice exhibited a decrease in CD24^+^GSIB4^−^ ciliated epithelial cells with HMPV infection, showing IFN-λ may be important for ciliated cell turnover (Fig. 5E). GSIB4^+^ basal epithelial cell upregulation during infection appeared to be both type I and III IFN-dependent, as this phenotype was lost in *Ifnar1*^−/−^ and *Ifnlr1*^−/−^ mice (Fig. 5F). Other myeloid and epithelial subpopulations tested showed no difference between HMPV-infected groups (Fig. S10). Collectively, these results indicate that type I IFN and IFN-λ drive differential innate immune cell recruitment, which reflects their distinct roles in the immune response to HMPV.

### IFN-λ is critical for limiting HMPV spread from upper to lower airways

**Fig 6:**
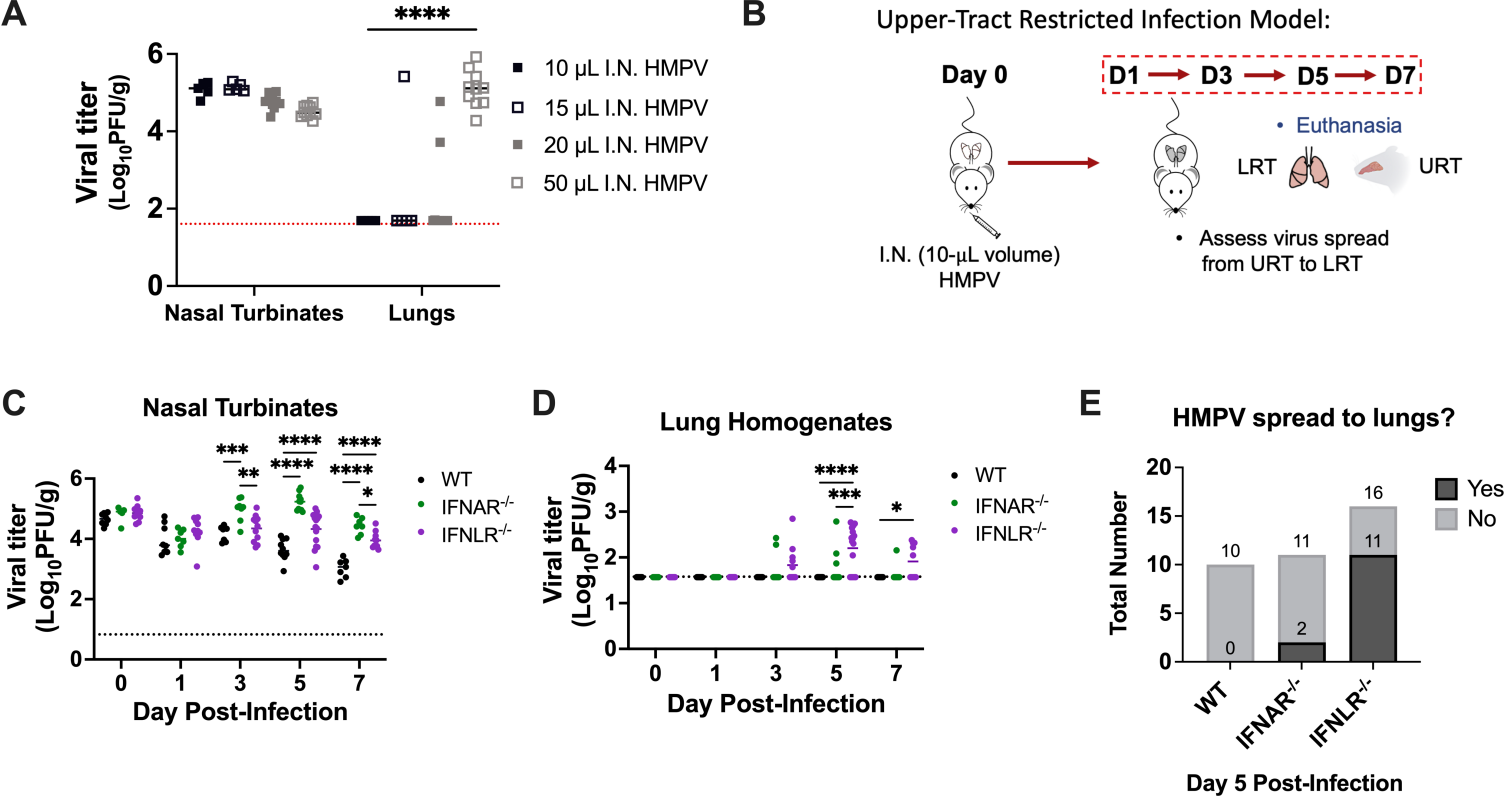
IFN-λ limits HMPV spread from upper to lower airways. (**A**) C57BL/6 (**B6**) mice were infected intranasally with 5 × 10^5^ PFU TN/94-49 HMPV in 10-, 15-, 20-, and 50 µL volumes (*n* = 10 mice per group). Mice were euthanized 5 min post anesthesia recovery. Lung and nasal turbinate viral titers (plaque-forming units/g) were quantified by plaque assay. (**B**) Schematic diagram of upper respiratory tract (URT)-restricted HMPV infection. (**C, D**) WT (shown in black), *Ifnar1*^−/−^ (labeled IFNAR^−/−^, shown in green), and *Ifnlr1*^−/−^ (labeled IFNLR^−/−^, shown in purple) mice were infected with the URT-restricted infection model of 10 µL of 5 × 10^5^ PFU TN/94-49 HMPV intranasally, and titer (PFU/g) was measured in lung homogenates (D) or nasal turbinates (C) by plaque assay. (**E**) Data from 6C and 6D graphically depicted to highlight lower airway HMPV spread on day 5 post-infection. Limit of detection was noted by dashed line. Analyses were done by two-way ANOVA. *P < 0.05, **P < 0.01, ***P < 0.001, ****P < 0.0001.

We previously found that mice lacking IFN-λ had reduced lung virus with high nasal burden (Fig. 4E and F). Thus, we tested whether IFN-λ directly restricted HMPV in the lungs vs affecting spread through the respiratory tract. Based on a prior model used in influenza (48), we developed an upper tract-only infection model of HMPV and found that a small (10 µL) volume delivered intranasally delivers HMPV solely to the upper airways and not lungs (Fig. 6A). We used this model to assess HMPV spread from upper to lower airways over time (Fig. 6B). None of the WT mice tested showed HMPV spread to lungs despite high upper airway titers (Fig. 6C and D). However, by day 5 post-infection, 11 of 16 (69%) *Ifnlr1*^−/−^ mice tested showed HMPV spread to lower airways (Fig. 6D and E). In contrast, only 2 of 11 (18%) *Ifnar1*^−/−^ mice showed lung HMPV burden. These data support a role for IFN-λ in restricting HMPV spread from upper to lower airways.

### Loss of IFN-λ signaling increases inflammatory cytokine and ISG levels

**Fig 7:**
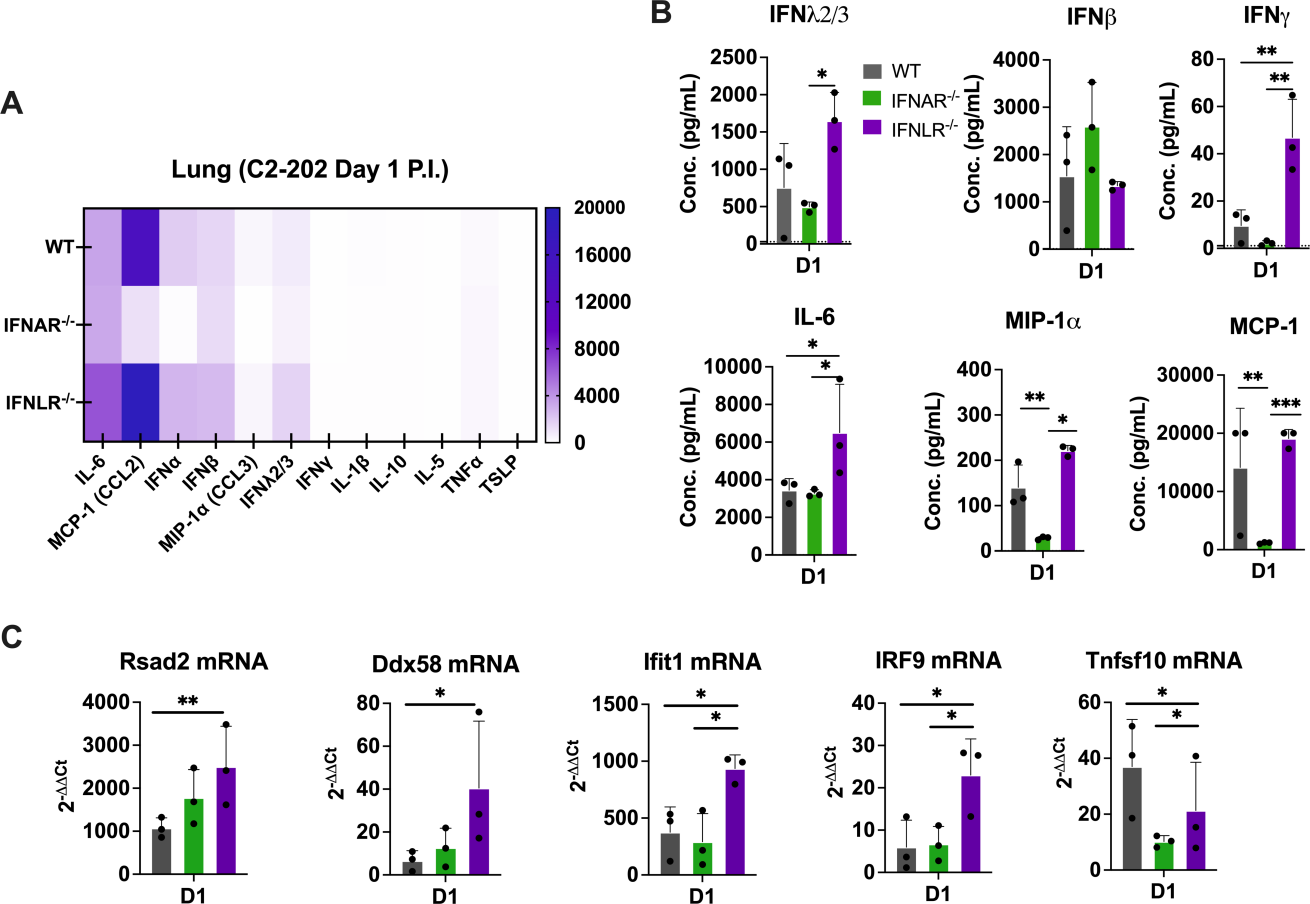
Inflammatory cytokine expression is altered by loss of IFN-λ signaling. WT (shown in black), *Ifnar1*^−/−^ (labeled IFNAR^−/−^, shown in green), and *Ifnlr1*^−/−^ (labeled IFNLR^−/−^, shown in purple) mice were infected with 5 × 10^5^ PFU C2-202 HMPV. (**A**) Protein expression levels by Luminex assay (ng/mL) of inflammatory cytokines in lung homogenates collected on day 1 post-infection from 5 × 10^5^ PFU C2-202 HMPV-infected mice graphically represented by heat map. (**B**) Protein expression levels by Luminex of IFN-λ2/3, IFN-β, IFN-γ, IL-6, MIP-1α, and MCP-1 cytokines in lung homogenate from WT, *Ifnlr1*^−/−^ (labeled IFNLR^−/−^), and *Ifnar1*^−/−^ (labeled IFNAR^−/−^) mice infected with 5 × 10^5^ PFU C2-202 HMPV and harvested day 1 post-infection. Data are shown in 7A and 7B from one representative Luminex plate (*n* = 3 mice per group) with similar findings on repeat assay. (**C**) qPCR analysis of interferon-stimulated genes was done for lung homogenate of mice described in 7B. Data are shown as mean ± standard deviation. Analyses were done by one-way ANOVA. *P < 0.05, **P < 0.01, ***P < 0.001.

Considering our findings of distinct roles for type I IFN and IFN-λ in controlling viral replication, we next tested whether type I IFN and IFN-λ promote differential expression of inflammatory cytokines. To do this, we used a multiplex bead-based immunoassay of mouse lung homogenates in WT, *Ifnar1*^−/−^, and *Ifnlr1*^−/−^ mice day 1 post-infection. *Ifnar1*^−/−^ mice had overall decreased cytokine expression in the lung compared to WT (Fig. 7A). In contrast, *Ifnlr1*^−/−^ mice showed higher lung expression of several cytokines compared to WT, including pro-inflammatory factors IFN-γ, IL-6, and macrophage/monocyte chemoattractants MIP-1α and MCP-1 (Fig. 7B). Importantly, compensatory effects, such as increased IFN-λ in *Ifnar1*^−/−^ mice or increased type I IFN (IFN-β) in *Ifnlr1*^−/−^ mice, were not observed (Fig. 7B). Using the same mouse lung homogenates, we also tested whether type I IFN and IFN-λ induced differential interferon-stimulated gene (ISG) expression. We selected ISGs to test based on prior studies of mouse and porcine intestinal epithelial cells that showed selective upregulation of IFN-λ-specific ISGs, including DDX58, Irf9, Rsad2, Ifit1, and Tnfsf10 (49, 50). These five antiviral ISGs were also upregulated in SARS-CoV-infected wild-type and *Ifnar1*^−/−^ mice, but not mice lacking IFN-λ signaling (51, 52). We measured expression of these five ISGs by qPCR and found that, while induction was not completely IFN-λ-specific, *Ifnlr1*^−/−^ mice showed elevated ISG expression comparable to and often higher than WT (Fig. 7C). Taken together, these findings suggest that IFN-λ signaling is important for limiting inflammatory cytokine and ISG induction, highlighting an immunomodulatory role for IFN-λ.

### IFN-λ prophylaxis and treatment reduce HMPV burden without driving inflammation

**Fig 8:**
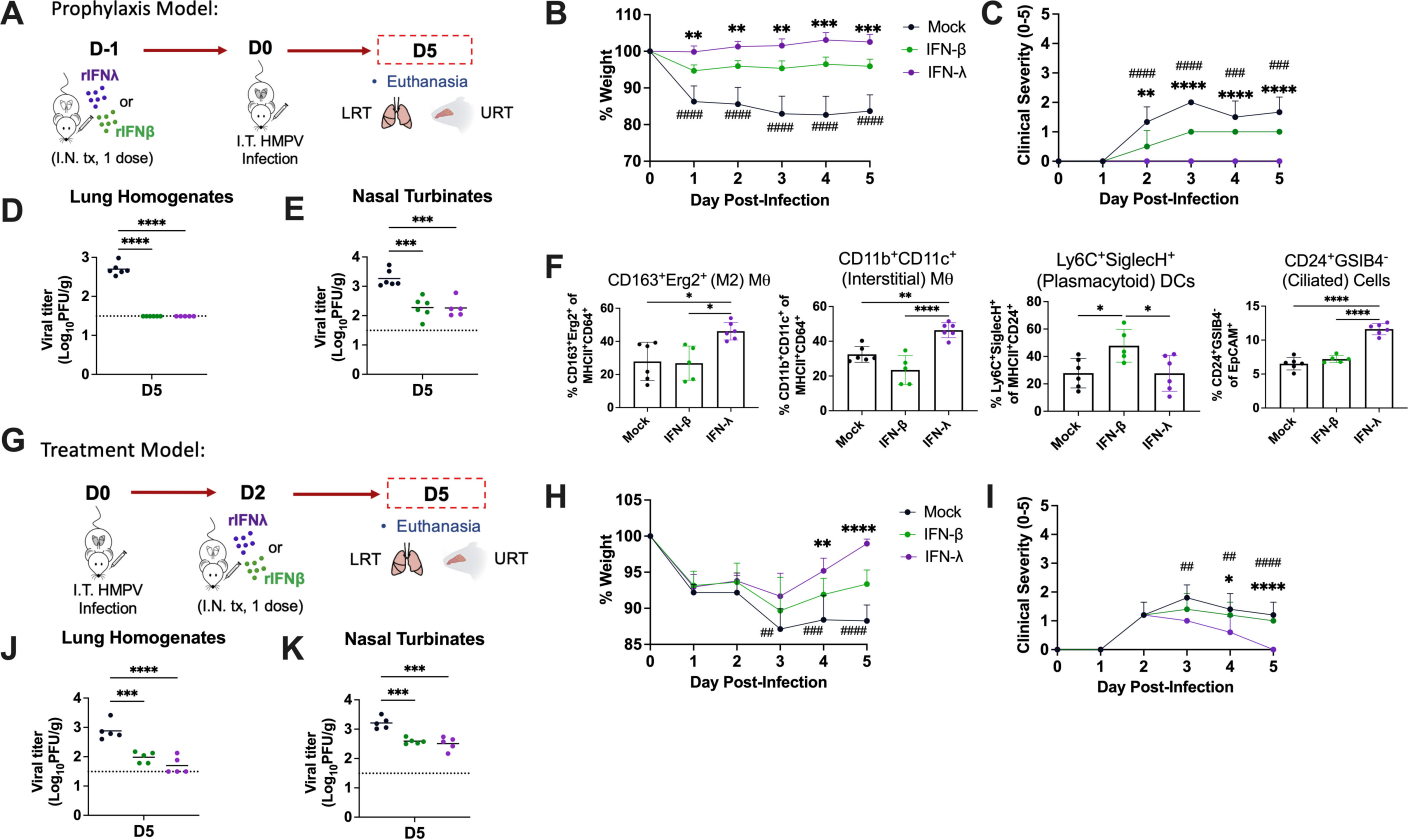
IFN-λ prophylaxis and treatment reduce HMPV burden without inflammation. C57BL/6 (**B6**) mice were infected with 5 × 10^5^ PFU C2-202 HMPV. (**A**) In a prophylaxis model (schematic diagram shown), recombinant IFN-λ (1 µg) or IFN-β (5 µg) was administered intranasally 1 days prior to infection. Mock prophylaxis included intranasal delivery of an equal volume of 0.1% bovine serum albumin (BSA). Mice receiving mock prophylaxis (shown in black), IFN-β prophylaxis (shown in green), and IFN-λ prophylaxis (shown in purple) were characterized (**B–F**). Disease was assessed by measuring body weight (**B**) and clinical severity scores (**C**) to day 7 post-infection. Weight represented as % of day 0. Clinical severity scores were measured by assigning 1 point out of 5 for each of the following criteria: hunching, huddling, fur ruffling, rapid breathing, and lethargy. Analyses were done by two-way ANOVA. ### *P* < 0.001, #### *P* < 0.0001 for mock vs both IFN-β and IFN-λ. **P < 0.01, ***P < 0.001, ****P < 0.0001 for IFN-λ vs IFN-β. (**D, E**) HMPV titer (PFU/g) was measured in lung homogenates (**D**) or nasal turbinates (**E**) of mice receiving IFN prophylaxis. (**F**) Frequency of lung innate immune and epithelial cell populations in mice receiving IFN prophylaxis euthanized day 1 post-infection by flow cytometry. (**G**) In a treatment model (schematic diagram shown), recombinant IFN-λ (1 µg) or IFN-β (5 µg) was administered intranasally 2 days post-infection. Mice receiving mock treatment (shown in black), IFN-β treatment (shown in green), and IFN-λ treatment (shown in purple) were characterized (**G–K**). Disease was assessed by measuring body weight (**H**) and clinical severity scores (**I**) to day 7 post-infection. Mock treatment included intranasal delivery of an equal volume of 0.1% BSA. (**J, K**) HMPV titer (PFU/g) was measured in lung homogenates (**J**) or nasal turbinates (**K**) of mice receiving treatment. Limit of detection noted by dashed line. Analyses were done by one-way ANOVA. *P < 0.05, **P < 0.01, ***P < 0.001, ****P < 0.0001.

Given evidence for IFN-λ in limiting HMPV replication and spread without contributing to lung inflammation, we tested the effects of IFN-λ therapy *in vivo* in HMPV-infected mice. We used two therapeutic models: a prophylaxis model, where IFN was given 1 day prior to infection, and a treatment model, where IFN was administered 2 days post-infection (Fig. 8A and G ). We first optimized type I IFN (IFN-β) and IFN-λ doses to maximize levels of ISG (MX1, IRF9) induction in mouse lungs (Fig. S11). In the prophylaxis model, both IFN-β and IFN-λ pre-treatment reduced weight loss and clinical disease (Fig. 8B and C). However, mice receiving IFN-β still showed 5%–10% weight loss and mild clinical disease, while mice receiving IFN-λ showed no weight loss or disease. Both IFN-β and IFN-λ pre-treatment abolished lung HMPV burden by day-5 post-infection and reduced nasal HMPV burden to similar degrees (Fig. 8D and E). Thus, IFN-λ promotes upper and lower airway HMPV clearance, but without the increased inflammatory pathogenesis observed with IFN-β treatment. We also examined which lung innate immune cell populations were altered by IFN-β or IFN-λ pre-treatment. Reciprocal changes were seen to IFN receptor knockout studies (Fig. 4), with IFN-λ promoting recruitment/retention of M2 macrophages, CD11b^+^CD11c^+^ interstitial macrophages, and ciliated epithelial cells while IFN-β promoted pDC recruitment by day 1 post-infection (Fig. 8F). Other lung myeloid and epithelial cell types at day 1 post-infection showed no observable differences (Fig. S12A through O). We also assessed lung cell populations in mice receiving IFN-β or IFN-λ without infection and observed recruitment of the same cell populations, confirming that these were IFN-driven changes (Fig. S12P and Q).

In the treatment model, both IFN-β and IFN-λ administration reduced weight loss and clinical disease although IFN-λ-treated mice showed slightly greater weight loss recovery and disease reduction (Fig. 8G through I). IFN-β and IFN-λ treatment also showed a similar decrease in lung and nasal HMPV burden (Fig. 8J and K), again confirming that IFN-λ promotes HMPV clearance and resolution of disease without increased inflammation. This was supported by lung histology, which show reduced pathology with both IFN-β or IFN-λ prophylaxis (Fig. S13A and C) or IFN-λ treatment (Fig. S13B) but continued mild inflammation in IFN-β-treated mouse lungs (Fig. S13C). Collectively, these findings suggest that IFN-λ therapy is advantageous in reducing HMPV burden and mitigating disease without contributing to inflammatory pathogenesis.

## DISCUSSION

Here, we uncover novel roles for IFN-λ in limiting lung HMPV replication and spread, impacting recruitment of lung macrophages and infection-driven loss of ciliated epithelial cells, and modulating expression of pro-inflammatory cytokines and ISGs. We also show that IFN-λ exerts antiviral effects against two strains of HMPV *in vitro* and that *in vivo* models of IFN-λ prophylaxis and clinical treatment reduce upper and lower tract viral burden without contributing to inflammatory disease. These findings suggest clinical utility for IFN-λ against HMPV infection.

Both type I IFN and IFN-λ showed similar degrees of protein expression by day 1 after HMPV infection. A study of lung epithelial cells infected with vesicular stomatitis virus also reported no difference in type I IFN and IFN-λ upregulation although noted delays in responses to IFN-λ (53). We also observed differential lung kinetics of type I IFN and IFN-λ, with type I IFN rapidly decreasing to undetectable by day 1 post-infection, while IFN-λ was detectable to day 7 post-infection. This supports previous findings of HMPV- and RSV-infected BALB/c mice that showed a drop in IFN-β levels in bronchoalveolar lavage fluid by 48 h post-infection while IFN-λ levels remained high (10).

Since we observed a robust and sustained production of IFN-λ post-infection, we next wanted to elucidate the primary cellular source of IFN-λ in our HMPV mouse model. Alveolar type II epithelial cells were main producers of IFN-λ *in vivo* in response to HMPV infection. Other studies also reported epithelial cells as primary IFN-λ producers in models of influenza, RSV, and reovirus infection, supporting our findings (13, 17, 18). We found that DCs, primarily cDC1 and pDC, were minor producers of IFN-λ in HMPV infection. Myeloid cells have been shown to produce IFN-λ in response to poly I:C (54). Respiratory epithelial cells and DC also showed high lung IFN-λ gene expression in influenza virus infection (37). scRNA-seq analysis revealed that the *Ifnlr1*^−/−^ subunit was expressed only on epithelial cells and neutrophils in HMPV-infected mouse lungs, suggesting these cell populations are mainly responding to IFN-λ. Analysis of HMPV transcripts showed internalized virus in mainly macrophages and to a lower degree in epithelial cells, but not dendritic cells, highlighting IFN-λ production by both HMPV-infected and non-infected lung cells. A study assessing lung co-localization of IFN-λ and SARS-CoV-2 similarly noted IFN-λ production by both non-infected and infected cell populations, similar to what we observe here (55).

Loss of IFN-λ signaling (*Ifnlr1*^−/−^ mice) led to reduced lung CD11b^+^CD11c^+^ interstitial macrophages in early HMPV infection, implicating IFN-λ in recruitment of lung macrophages. Macrophages are responsive to IFN-λ, with IFN-λ stimulating macrophage production of ISGs and cytokines that mediate immune cell chemotaxis (56). Interestingly, wild-type and *Ifnar1*^−/−^ mice, but not *Ifnlr1*^−/−^ mice, showed a decrease in lung ciliated epithelial cells early post-infection, suggesting IFN-λ may also drive this phenotype. This was surprising given the well-validated function of IFN-λ in promoting barrier integrity of epithelial cells (57–59). Two other studies show IFN-λ delays lung epithelial cell proliferation post-infection with influenza virus and SARS-CoV-2 (60, 61). However, we found that IFN-λ treatment increased lung ciliated epithelial cell populations, supporting other studies showing IFN-λ promotes expression of tissue repair factors (62). Thus, we hypothesize the early decrease in lung-ciliated cells may be a protective function of IFN-λ to maintain barrier integrity; however, further studies are needed to characterize the role of IFN-λ in epithelial repair and proliferation in HMPV infection.

We found that IFN-λ limits lung HMPV replication without contributing to inflammatory disease and that this phenotype was primarily mediated through CD45^−^ non-immune cell-mediated mechanisms. Additionally, we uncovered airway site-specific functions of IFNs, with IFN-λ alone limiting lung HMPV replication while both type I IFN and IFN-λ were important for limiting nasal HMPV replication. Differences in IFN expression based on location were also observed in patients with COVID-19, with IFN-λ increased in upper airway with mild disease but upregulated in lower tract with severe disease (63). IFN-λ, but not type I IFN, signaling was critical in restricting HMPV spread from upper to lower airways, similar to what has been previously reported for influenza (48). We also observed a potential modulatory role for IFN-λ in HMPV disease, as *Ifnlr1*^−/−^ mice showed higher expression of inflammatory cytokines and ISGs early post-infection. This was noted with influenza virus, where *Ifnlr1*^−/−^ mice showed greater lung levels of cytokines including IL-6, IFN-γ, and macrophage chemoattractants (37). IFN-λ has been linked to immunomodulatory functions of DCs, including the ability to promote adaptive antiviral Th1 and modulatory Treg responses (64). More studies are needed to define IFN-λ contributions to adaptive immunity in HMPV infection.

Ultimately, both models of IFN-λ prophylaxis and treatment showed less weight loss and clinical disease, decreased lung and nasal HMPV burden, and reduced lung immunopathology. Meanwhile, type I IFN administration reduced HMPV titers but showed less improvement of weight loss and higher lung histopathology scores suggesting a greater contribution to inflammatory disease. A mouse model of SARS-CoV-2 infection also showed decreased lung viral burden with intranasal delivery of IFN-λ as prophylaxis or treatment (55). Given increasing evidence for IFN-λ as an antiviral cytokine with minimal contribution to inflammation, phase II and III clinical trials of pegylated IFN-λ therapy have been done in patients with COVID-19 (65, 66). Those who received IFN-λ had lower SARS-CoV-2 load by day 7 post-infection, particularly patients with a high initial virus burden, and similar incidence of adverse events as those receiving placebo. Our data suggest we may see similar clinical success of IFN-λ treatment in HMPV infection, an acute and often severe respiratory illness with no current antiviral therapies available.

Our findings reveal novel protective antiviral and immunomodulatory functions of IFN-λ in HMPV infection, highlighting an important innate cytokine with therapeutic potential for patients with severe HMPV disease.

## Data Availability

All data are available from the corresponding author upon request. Single cell RNA sequencing data used in this paper are publicly accessible through Gene Expression Omnibus accession number GSE261511.

## SUPPLEMENTAL MATERIAL

Supplemental figures mbio.00550-24-s0001.pdf Fig. S1 through S6.

Additional supplemental figures mbio.00550-24-s0002.pdf Fig. S7 through S13.

Legends mbio.00550-24-s0003.docx Supplemental figure legends.

Table S1 mbio.00550-24-s0004.docx Antibody table.

ASM does not own the copyrights to Supplemental Material that may be linked to, or accessed through, an article. The authors have granted ASM a non-exclusive, world-wide license to publish the Supplemental Material files. Please contact the corresponding author directly for reuse.
