# Supplementary figures and images for: IFN-λ drives distinct lung immune landscape changes and antiviral responses in human metapneumovirus infection
Source: mBio. 2024 Mar 26;15(5):e00550-24. doi: 10.1128/mbio.00550-24 (PMC11077986; doi:10.1128/mbio.00550-24)

**Figure S1**

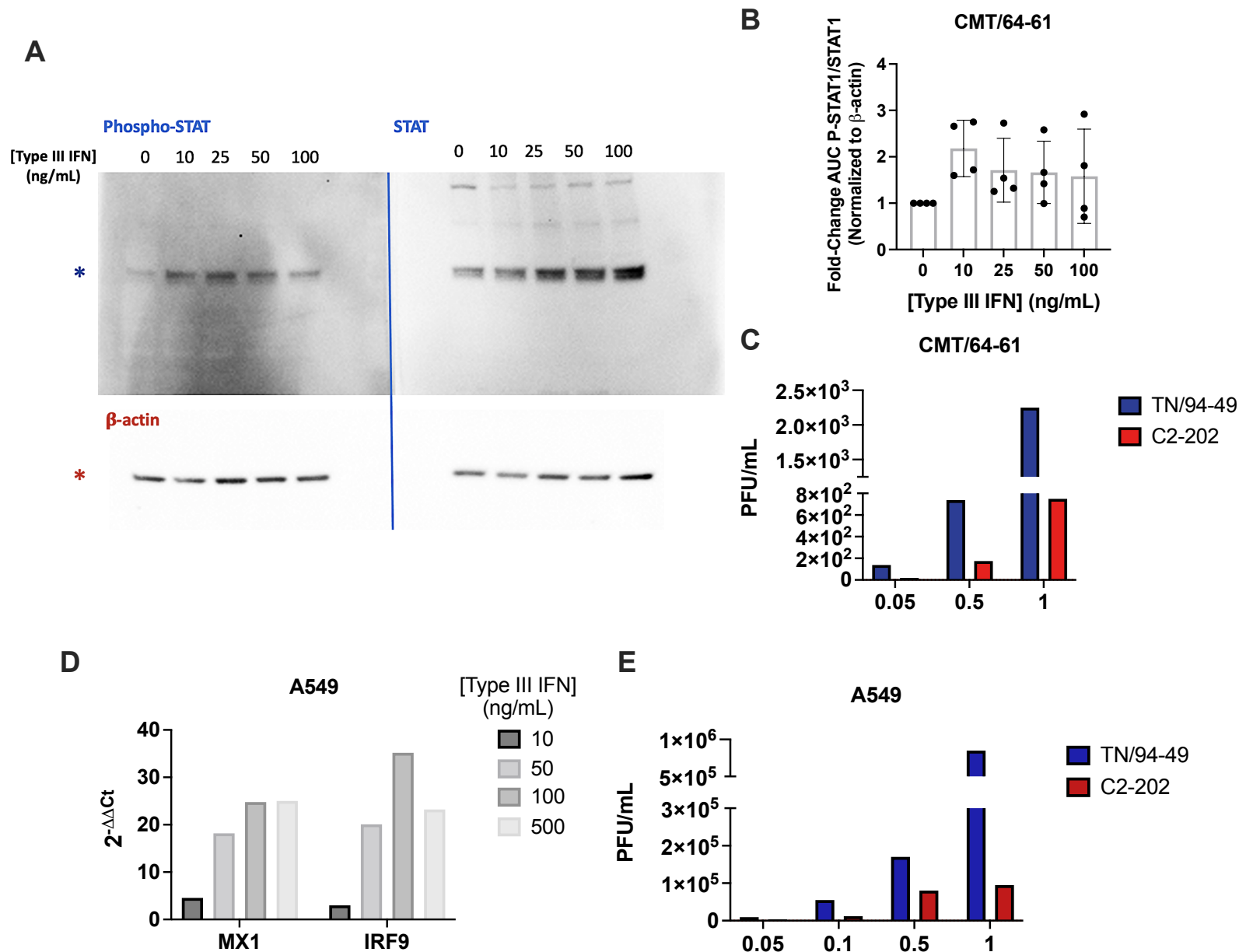

Figure S2

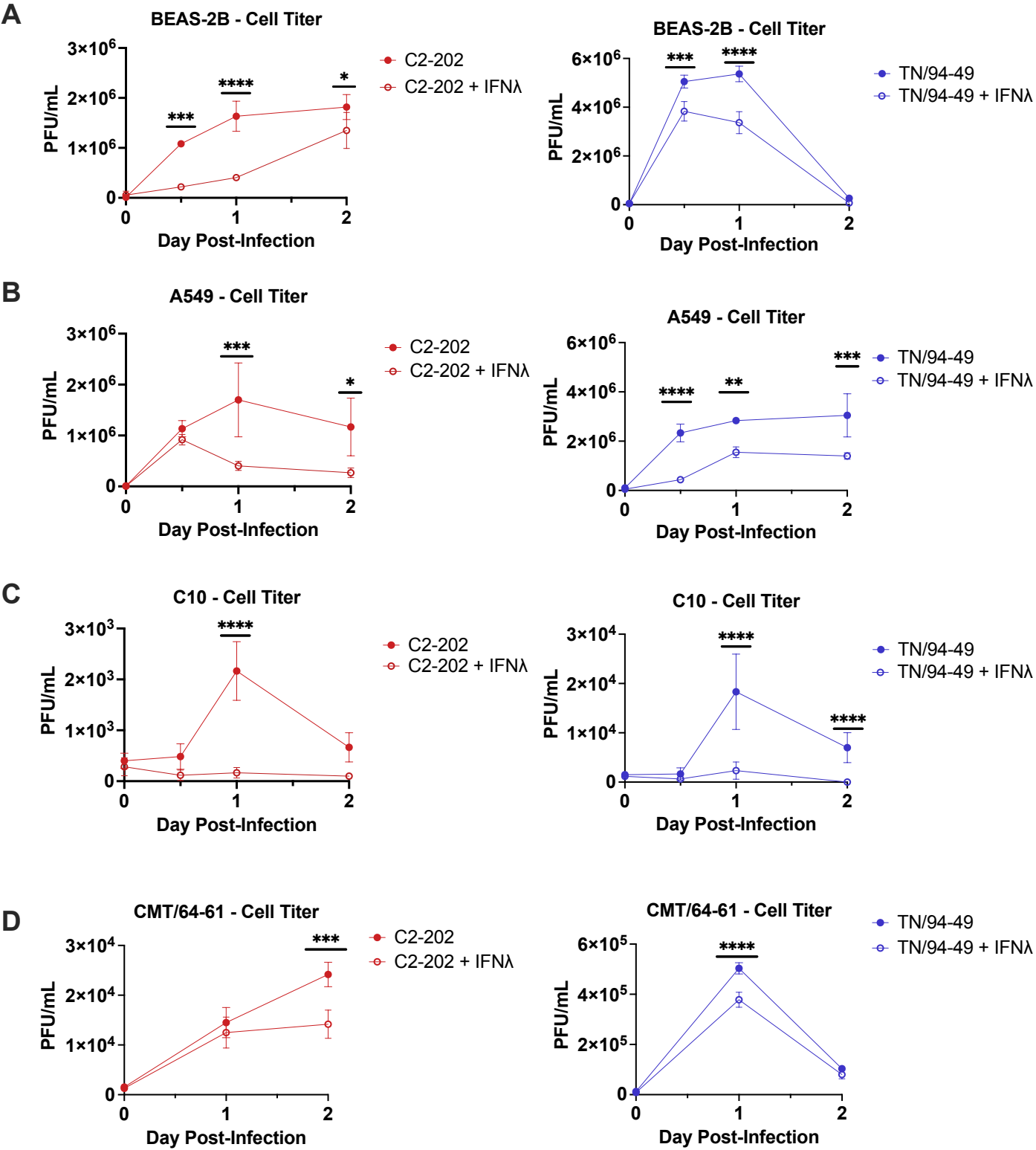

Figure S3

A

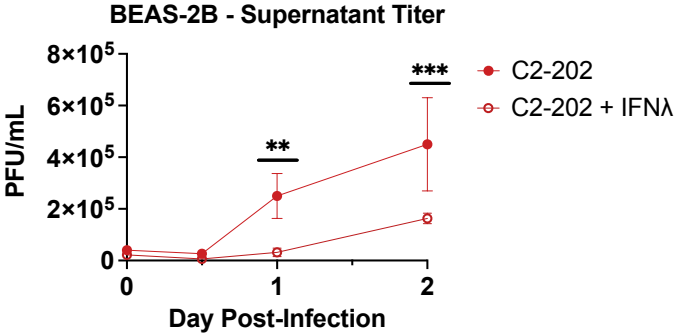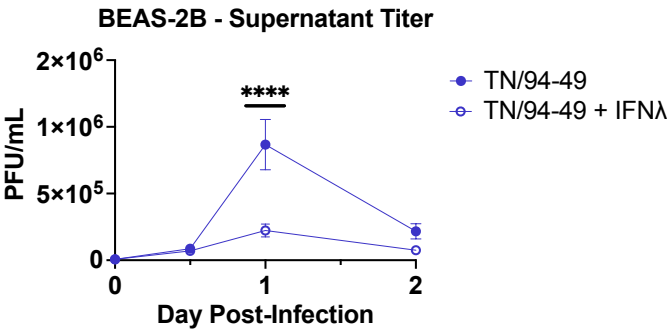

B

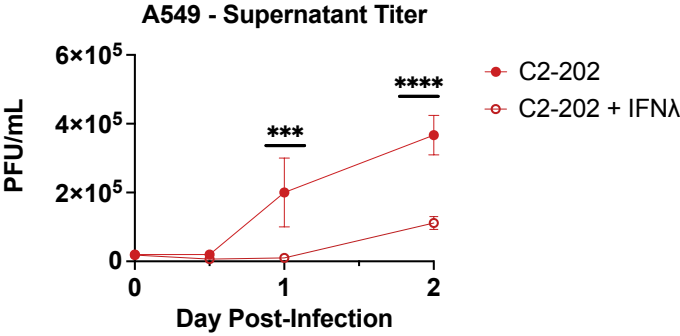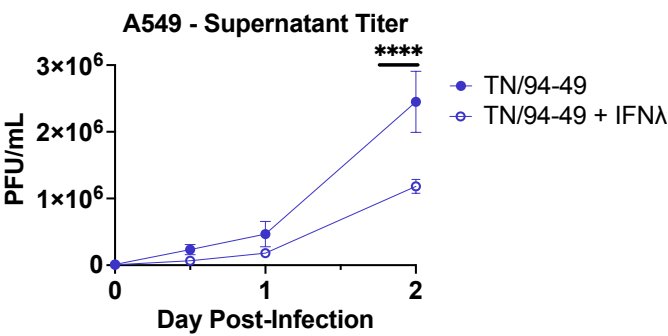

C

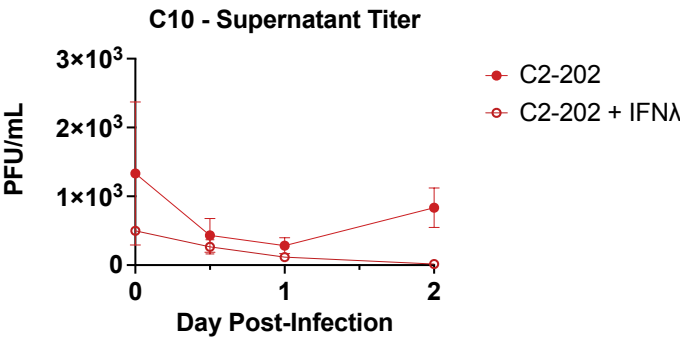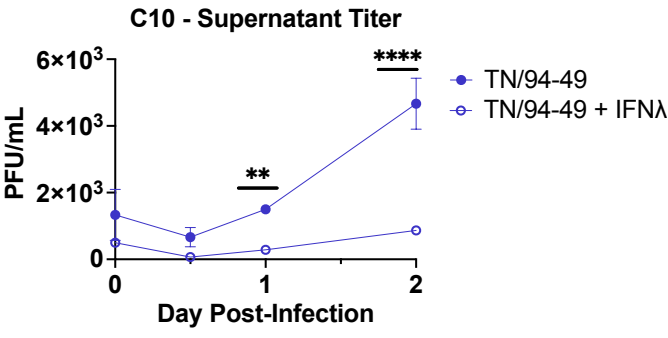

D

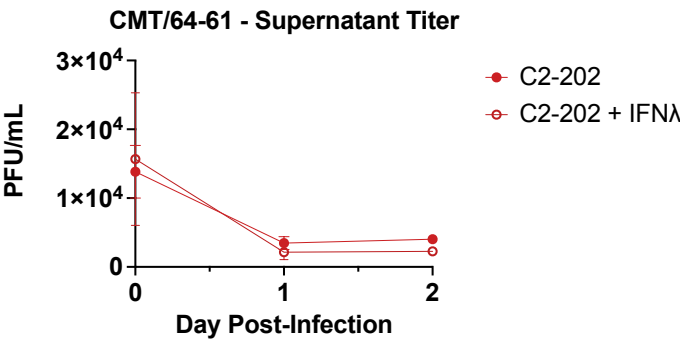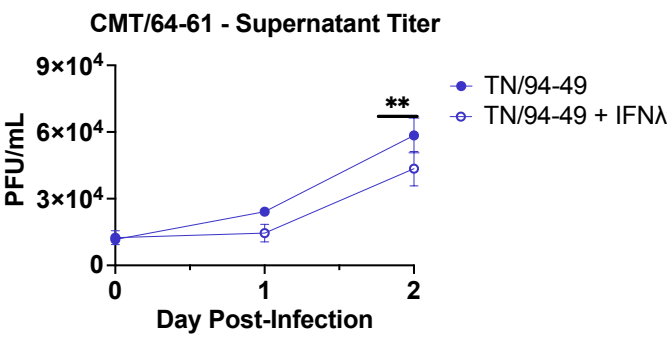

Figure S4

A

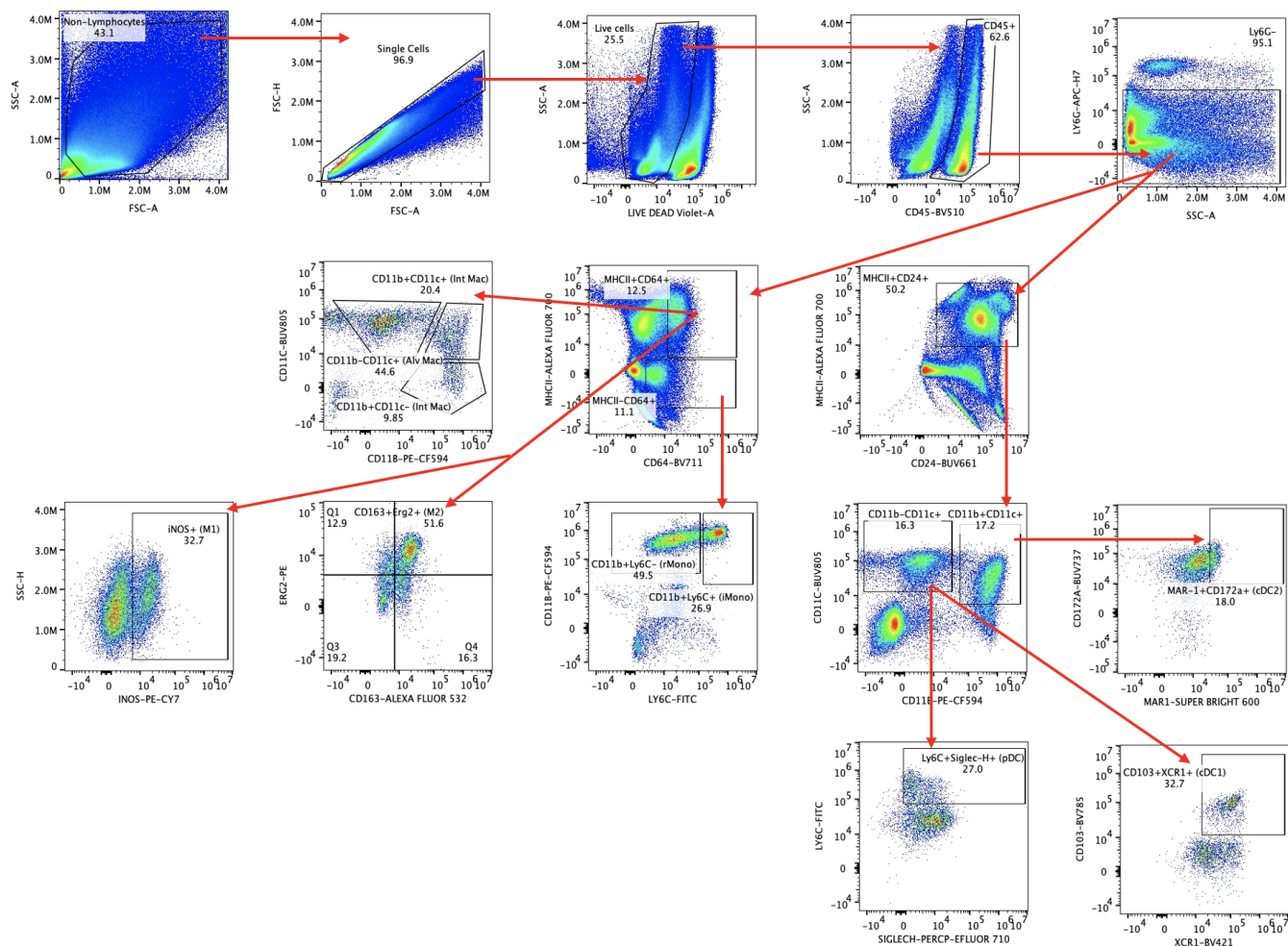

B

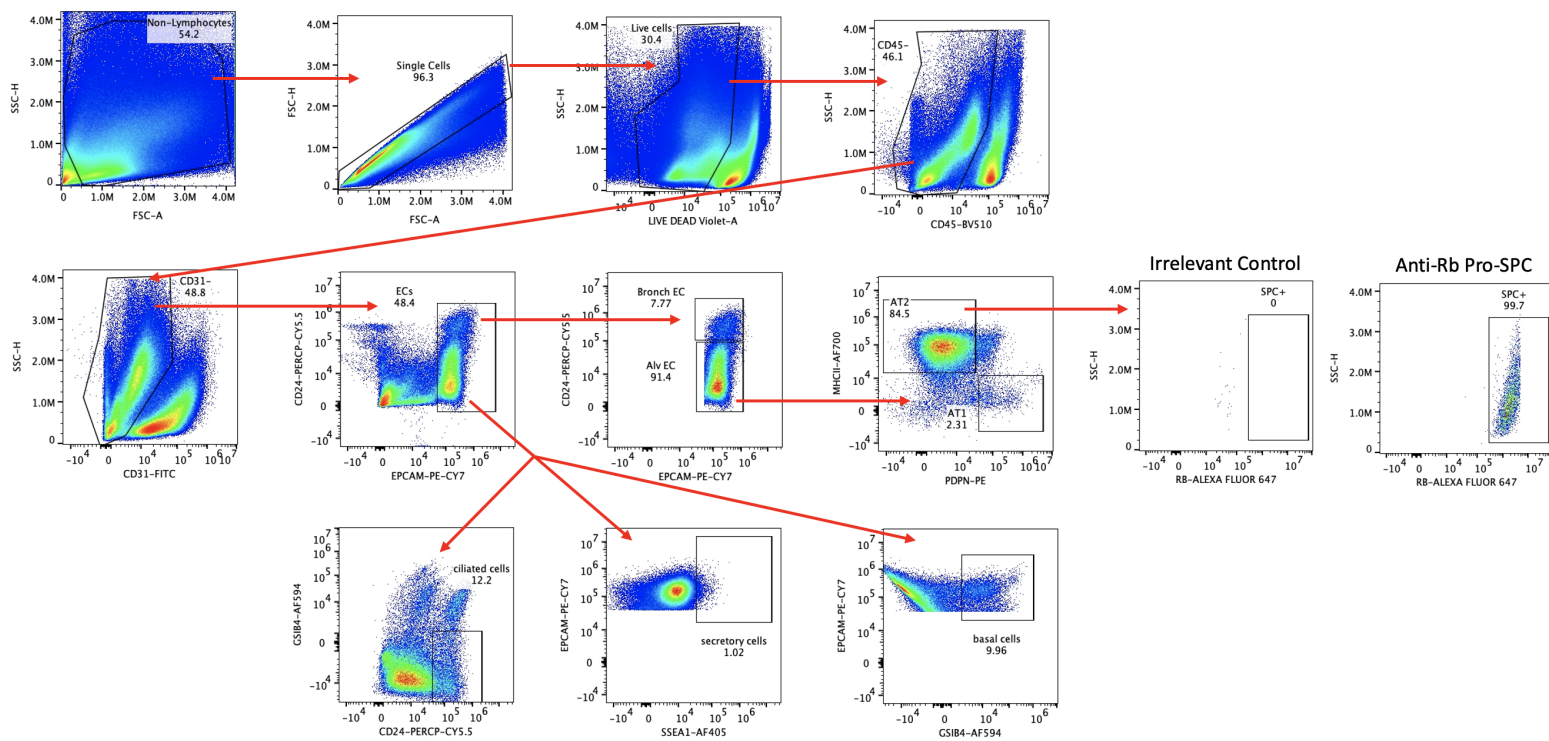

**Figure S5**

**A**

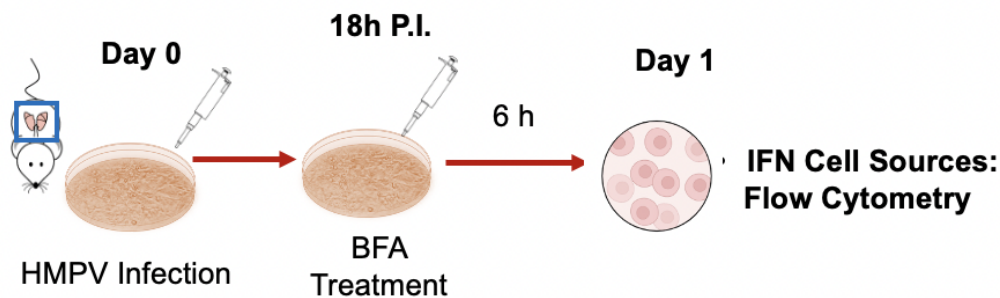

**B**

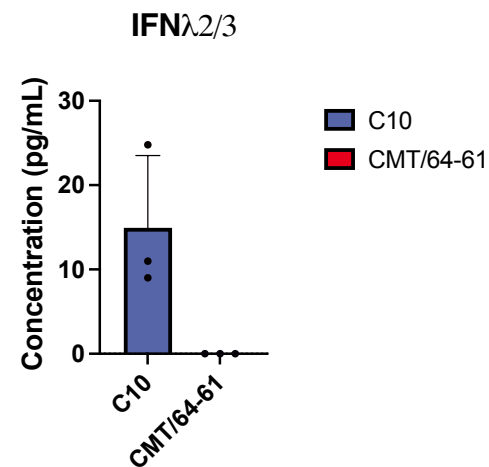

**C**

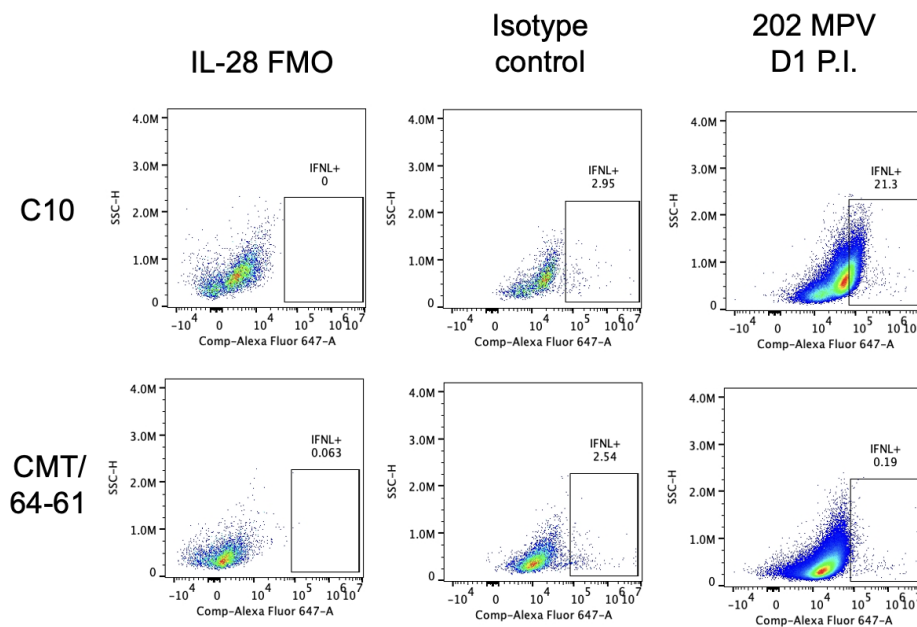

# Figure S6

**A**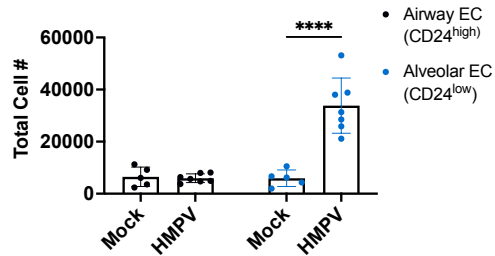**B**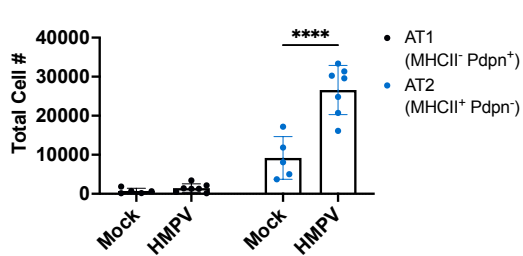**C**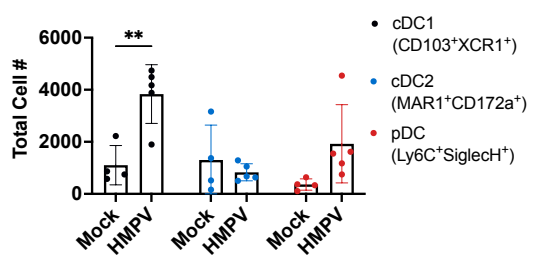**D**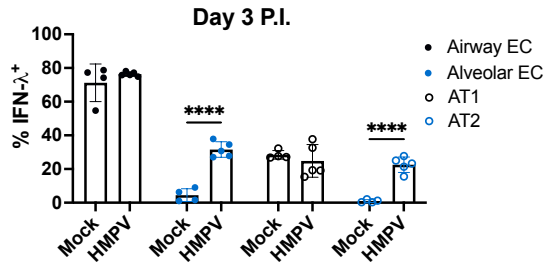**E**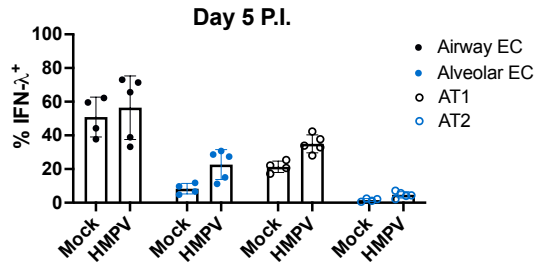

Supplement: Supplemental figures — Fig. S1 through S6. [file mbio.00550-24-s0001.pdf]

**Figure S7**

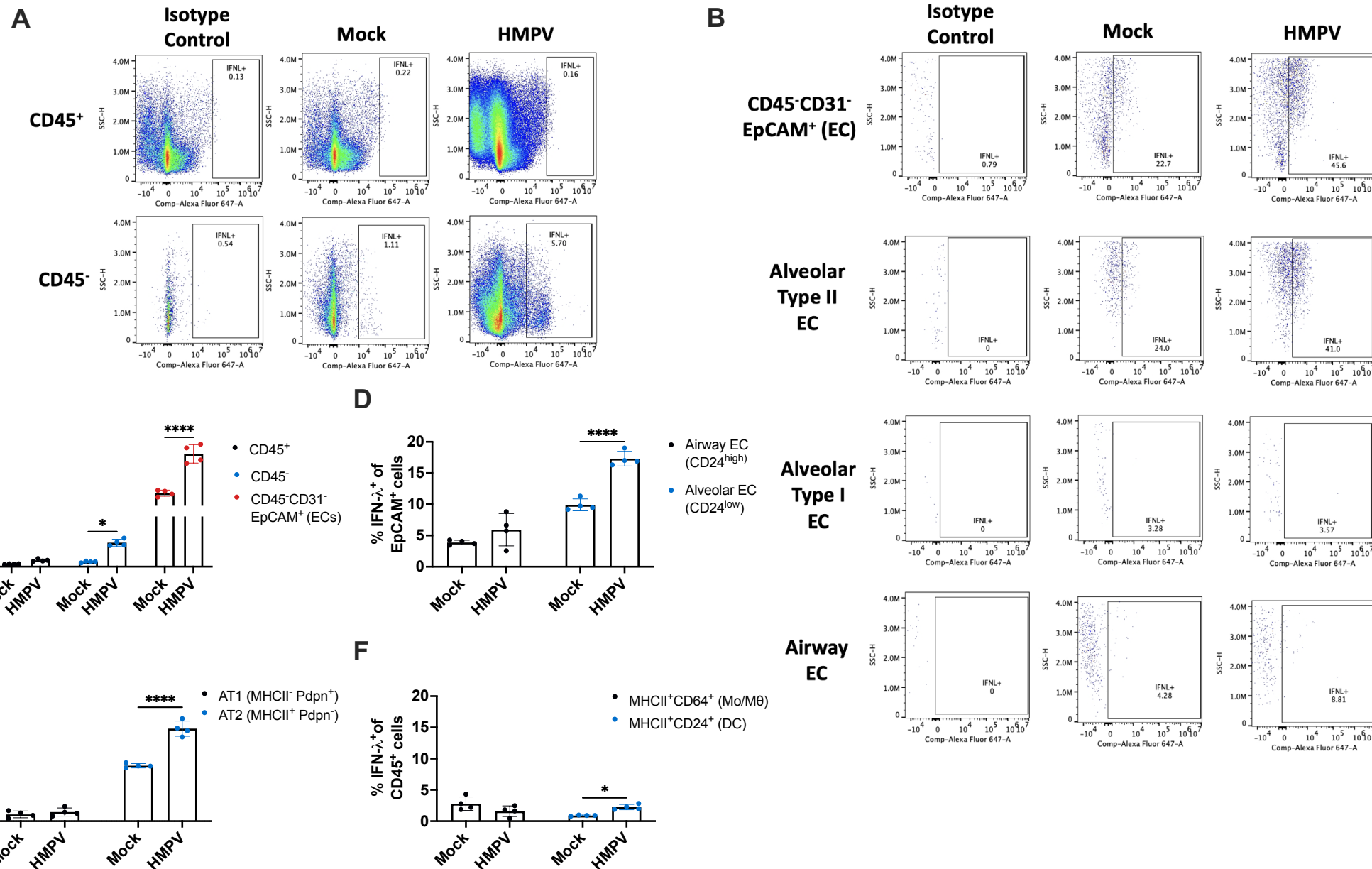

**Figure S8**

**A**

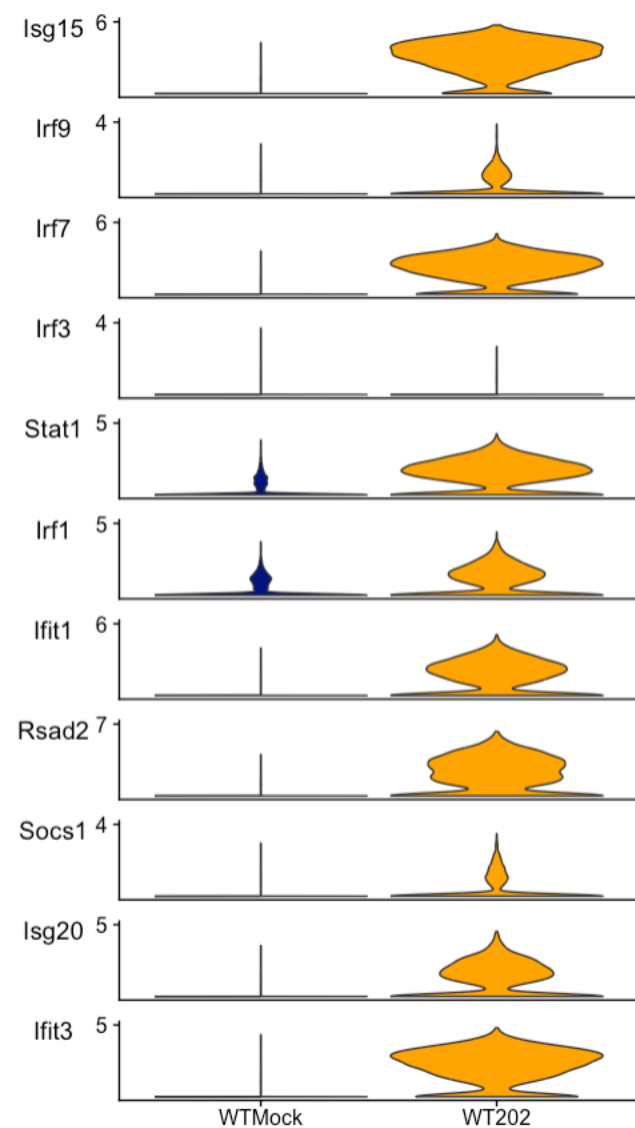

**B**

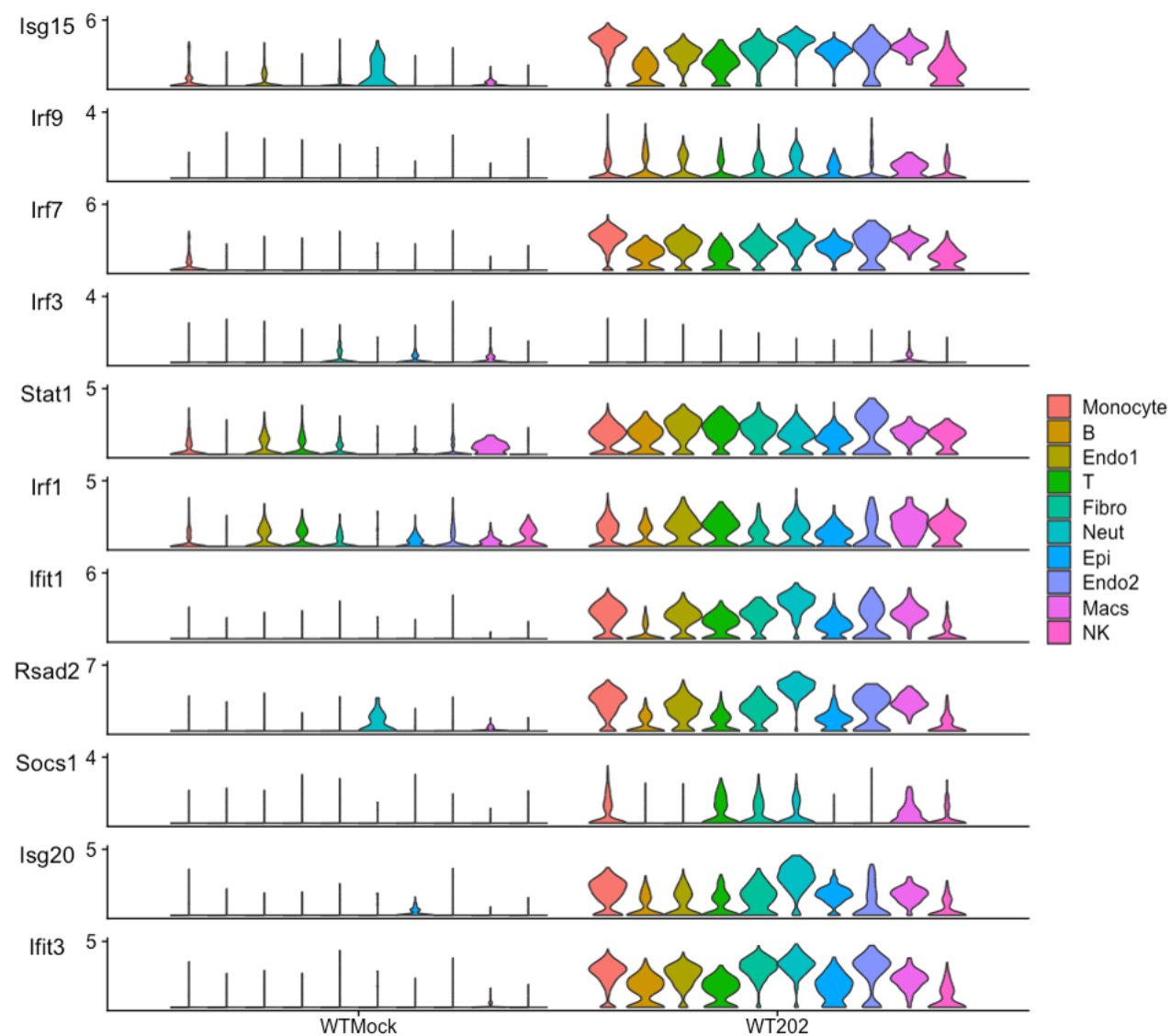

**Figure S9**

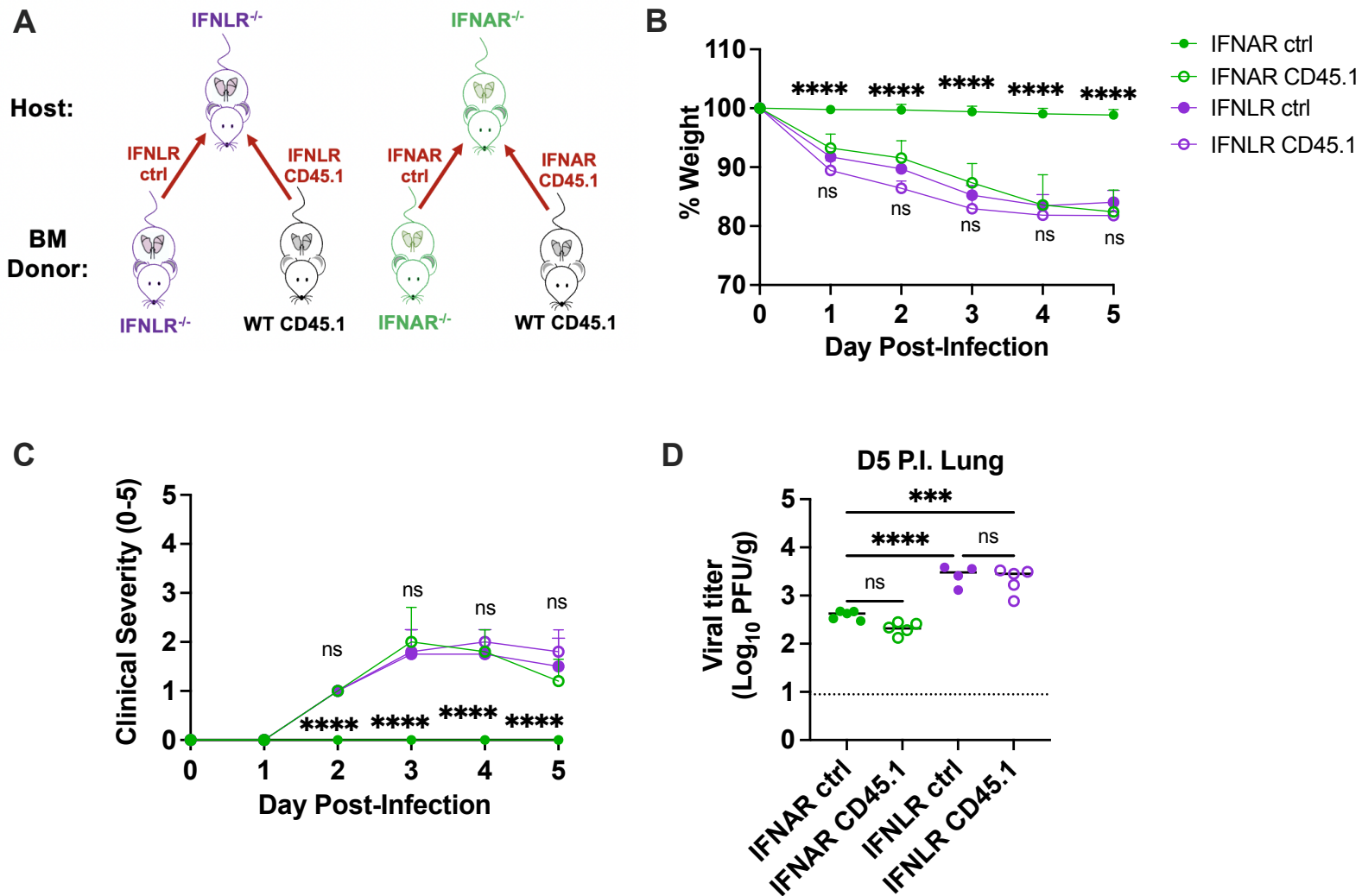

# Figure S10

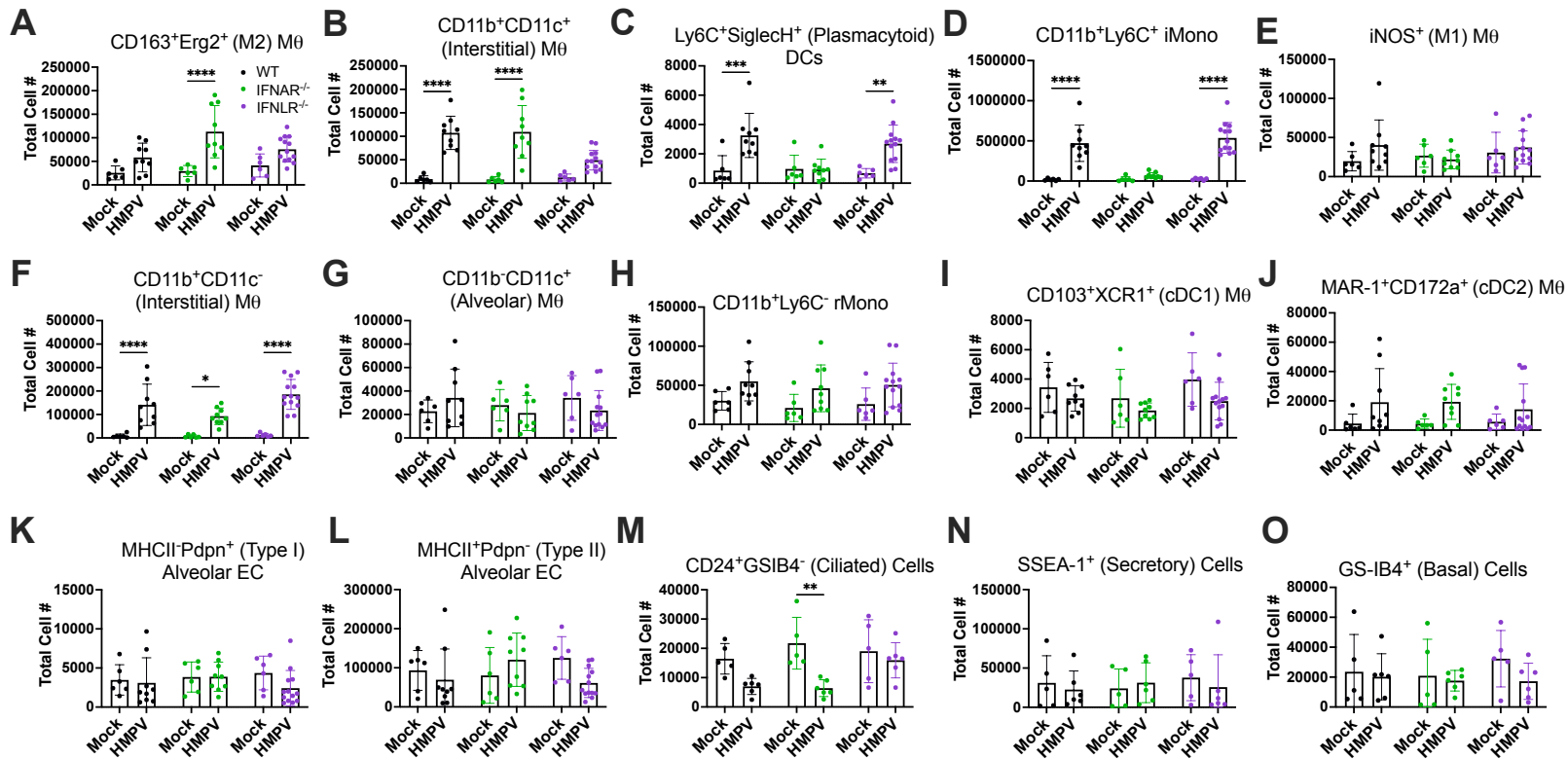

**Figure S11**

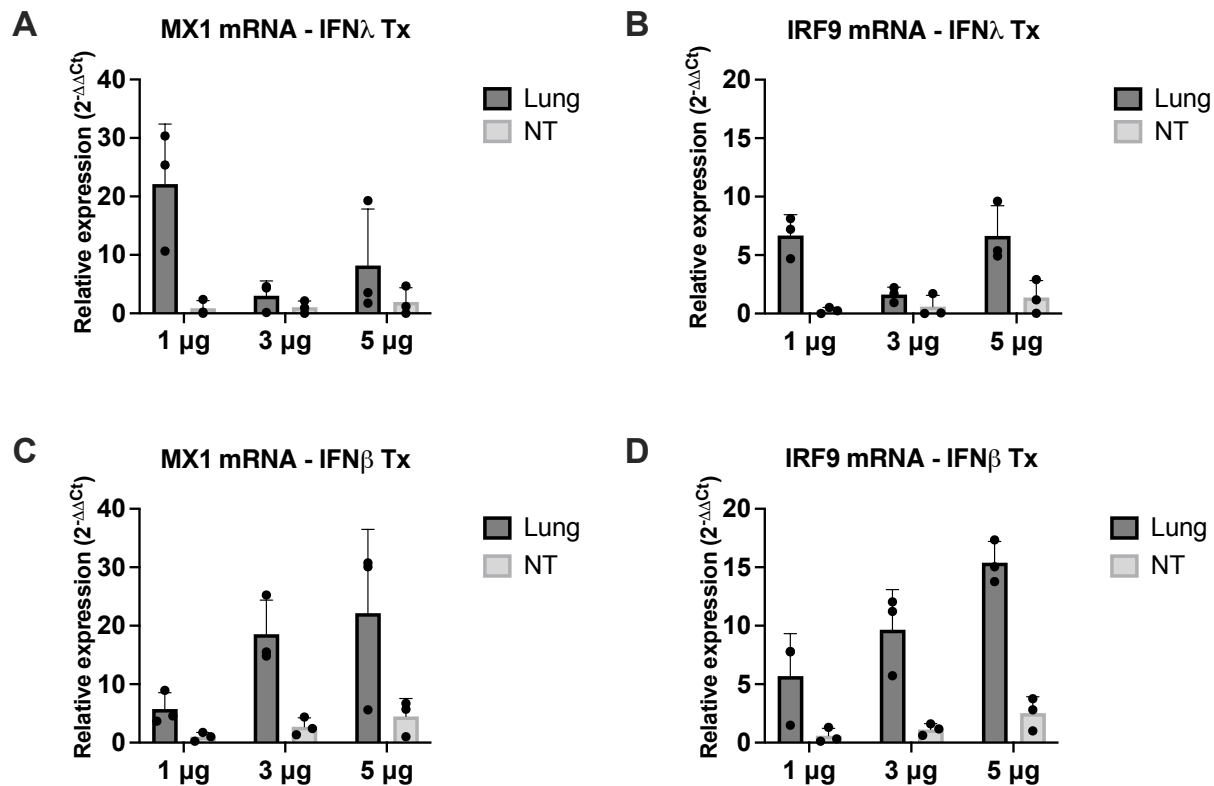

# Figure S12

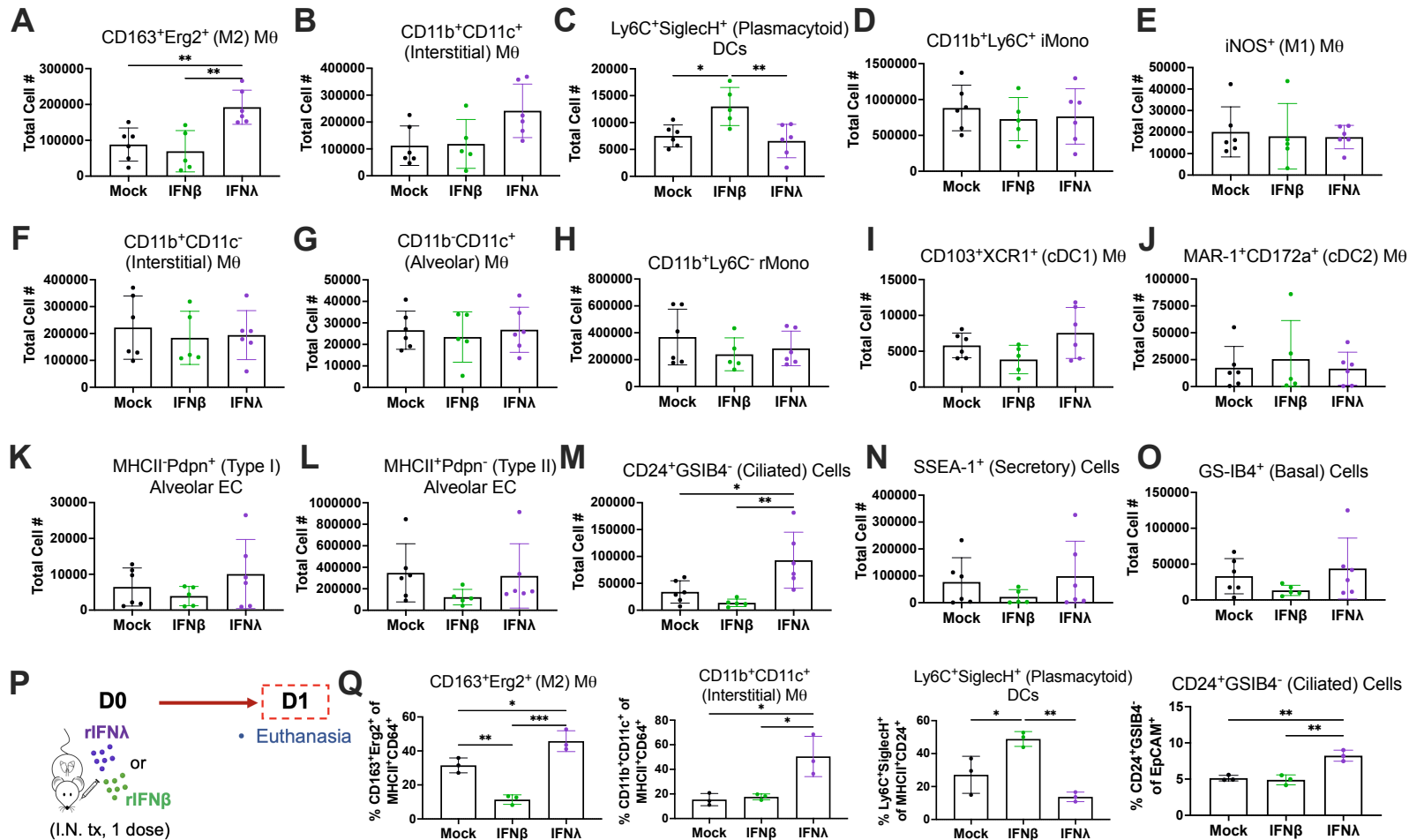

Figure S13

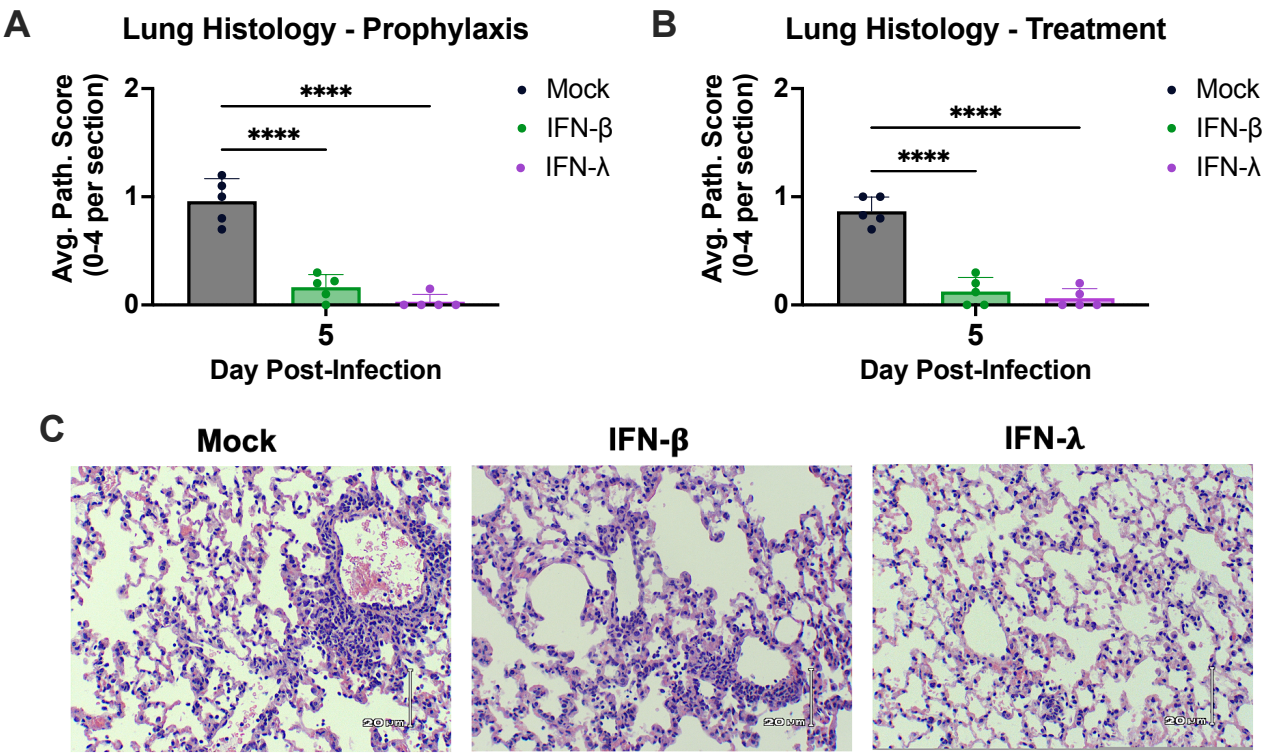

Supplement: Additional supplemental figures — Fig. S7 through S13. [file mbio.00550-24-s0002.pdf]
