# Supplementary material for: IFN-λ drives distinct lung immune landscape changes and antiviral responses in human metapneumovirus infection
Source: mBio. 2024 Mar 26;15(5):e00550-24. doi: 10.1128/mbio.00550-24 (PMC11077986; doi:10.1128/mbio.00550-24)
Supplement: Legends — Supplemental figure legends. [file mbio.00550-24-s0003.docx]

**Figure S1. Optimization of *in vitro* IFN-λ treatment models.** A-C) IFN dose and HMPV inoculum were optimized for mouse lung epithelial cells (CMT/64-61, C10). Representative optimization for CMT/64-61 cells is shown. A) For IFN dose optimization, STAT1 phosphorylation was measured in CMT/64-61 cells treated with various IFN-λ doses and harvested 30m later. B) Western blots described in S1A were quantified. Data is reported as ratio area-under-curve of phosphoSTAT1/total STAT1 normalized to β-actin housekeeping gene. C) For HMPV inoculum optimization, plaque assay quantitation was done for CMT/64-61 cells infected with TN/94-49 or C2-202 HMPV at various MOIs and harvested 1-hour post-inoculation. D-E) IFN dose and HMPV inoculum optimization for human lung epithelial cells (A549, BEAS-2B). Representative optimization for A549 cells is shown. D) For IFN dose optimization, qPCR analysis was done for interferon-stimulated genes MX1 and IRF9 in A549 cells treated with IFN-λ doses and harvested 24-hours later. Data is normalized to HPRT1 housekeeping gene and the null condition of mock-treated cells by the 2^-∆∆Ct^ method. E) For HMPV inoculum optimization, plaque assay quantitation was done for A549 cell lysate infected with HMPV at various MOIs and harvested 1-hour post-inoculation.

**Figure S2.** **IFN-λ treatment reduces HMPV intracellular titers in lung epithelial cells.** BEAS-2B (A), A549 (B), C10 (C), and CMT/64-61 (D) epithelial cells were infected with C2-202 (right) or TN/94-49 (left) HMPV, and plaque assay quantitation of cell lysate harvested 0-2 days post-infection was done. Cells received either human recombinant IFN-λ1 (BEAS-2B, A549) or mouse recombinant IFN-λ2 (C10, CMT/64-61) 1 day prior to HMPV infection. IFN-λ treatment doses and HMPV inoculum were optimized as described in Fig.S1. MOIs of 0.5 for TN/94-49 and 1 for C2-202 were used for CMT/64-61 infection. MOIs of 0.1 for TN/94-49 and 1 for C2-202 were used for C10 infection. MOIs of 0.1 for TN/94-49 and 0.5 for C2-202 were used for A549 infection. MOIs of 0.5 for TN/94-49 and 1 for C2-202 were used for BEAS-2B infection. 10 ng/mL of mouse IFN-λ2 were used for CMT/64-61 and C10 treatment. 100 ng/mL of human IFN-λ1 was used for A549 treatment. 500 ng/mL human IFN-λ1 was used for BEAS-2B treatment. Data is shown as mean ± standard deviation. Analyses are done by two-way ANOVA. * p<0.05, ** p<0.01, *** p<0.001, **** p<0.0001.

**Figure S3. IFN-λ treatment reduces HMPV extracellular titers in lung epithelial cells.** BEAS-2B (A), A549 (B), C10 (C), and CMT/64-61 (D) epithelial cells were infected with C2-202 (right) or TN/94-49 (left) HMPV, and plaque assay quantitation of supernatant harvested 0-2 days post-infection was done. Cells received either human recombinant IFN-λ1 (BEAS-2B, A549) or mouse recombinant IFN-λ2 (C10, CMT/64-61) 1 day prior to HMPV infection. IFN-λ treatment doses and HMPV inoculum are described in Figure S2. Data is shown as mean ± standard deviation. Analyses are done by two-way ANOVA. * p<0.05, ** p<0.01, *** p<0.001, **** p<0.0001.

**Figure S4. Gating strategies for mouse lung innate immune populations.** A) Flow cytometric gating strategy for mouse lung myeloid cells. We assessed frequency of macrophage, monocyte, and dendritic cell subpopulations. B) Flow cytometric gating strategy for mouse lung epithelial cells. We assessed frequency of airway and alveolar cells. Alveolar type II differentiation was validated by control gating for specific type II marker pro-surfactant protein C.

**Figure S5. Confirmation of IFN-λ staining in mouse lung epithelial cells.** A) IFN-λ staining was validated *in vitro* by flow cytometry. B) ELISA of IFN-λ levels shows that C10 cells, but not CMT/64-61 cells, express IFN-λ 24 hours post-infection with C2-202 HMPV. C) Staining of C10 or CMT/64-61 cells with mouse IL-28 (IFN-λ)-AF647 antibody day 1 post-infection (right) shows positive IFN-λ staining in C10 cells alone. Controls include no IL-28 antibody (left) or an isotype control of RORγT, a marker not expressed in epithelial cells, conjugated to the same fluorophore (middle).

**Figure S6. IFN-λ production by alveolar type II cells rapidly declines after early infection.** Mice were infected with 5x10^5^ PFU of HMPV strain C2-202 or mock cell lysate and IFN-λ expression in mouse lungs was measured. We quantified total number of IFN-λ2/3^+^ cells in alveolar vs. airway epithelial cell types of mouse lungs harvested day 1 post-infection (A). We also quantified total number of IFN-λ2/3^+^cells in alveolar cell subpopulations (B) and dendritic cell subpopulations (C) in lungs of mice described above. D-E) To assess IFN-λ production over time, we measured frequency of IFN-λ2/3^+^ cells in airway epithelial cells, alveolar cells, and alveolar subpopulations day 3 (D) or day 5 (E) post-infection with 5x10^5^ PFU C2-202. Data shown as mean ± standard deviation. Analyses done by two-way ANOVA. * p<0.05, ** p<0.01, *** p<0.001, **** p<0.0001.

**Figure S7. PrimeFlow RNA assay shows highest *Ifnl3* mRNA expression in alveolar type II cells.** Mice were infected with 5x10^5^ PFU of HMPV strain C2-202 or mock cell lysate and *Ifnl3* mRNA expression in mouse lungs was measured by PrimeFlow assay day 1 post-infection. A) Gating for *Ifnl3* mRNA expression is shown for CD45^+^ (immune) vs. CD45^-^ (non-immune) populations. B) Gating for *Ifnl3* mRNA expression is shown for epithelial cell subsets. C) Frequency of *Ifnl3*^+^ cells in CD45^+^ (immune) vs. CD45^-^ (non-immune) populations. D) Frequency of *Ifnl3*^+^ cells in alveolar vs. airway epithelial cell types. E) Frequency of *Ifnl3*^+^ cells in alveolar cell subpopulations in lungs of mice, showing upregulation of IFN-$\lambda$ in type II alveolar epithelial cells with HMPV infection. F) Frequency of *Ifnl3*^+^ cells in parent populations of macrophages vs. dendritic cells. Data shown as mean ± standard deviation. Analyses done by two-way ANOVA. * p<0.05, ** p<0.01, *** p<0.001, **** p<0.0001.

**Figure S8. Early HMPV infection induces broad interferon-stimulated gene upregulation.**

Single cell RNA sequencing (scRNA-seq) was performed on cells isolated from lungs of mice infected with 5x10^5^ PFU C2-202 HMPV or mock lysate and harvested day 1 post-infection. A) Total lung expression of various interferon-stimulated genes (ISGs) was measured. B) Expression levels of the ISGs in S8A were analyzed and stratified by cell type.

**Figure S9.** **IFN-λ control of lung HMPV replication is mediated by CD45^-^ non-immune cells.** A) In a bone marrow transplant model (schematic diagram shown), *Ifnar1*^-/-^ and *Ifnlr1*^-/-^ recipient mice were irradiated and given donor bone marrow from either wild-type B6 (CD45.1) mice (called either IFNLR or IFNAR CD45.1) or donor *Ifnar1*^-/-^ or *Ifnlr1*^-/-^ as controls (called either IFNLR or IFNAR CD45.1). Disease was assessed by measuring body weight (B) and clinical severity scores (C) to day 5 post-infection. Weight represented as % of Day 0. Clinical severity scores were measured by assigning 1 point out of 5 for each of the following criteria: hunching, huddling, fur ruffling, rapid breathing, and lethargy. Analyses done by two-way ANOVA. **** p<0.0001 for IFNAR ctrl vs. IFNAR CD45.1. ns (not significant) for IFNLR ctrl vs. IFNLR CD45.1. D) HMPV titer (PFU/g) was measured in lung homogenates of mice receiving IFN prophylaxis. Analyses done by one-way ANOVA. *** p<0.001, **** p<0.0001.

**Figure S10. Lung innate populations are altered by loss of IFN-λ or type I IFN signaling.** WT, *Ifnlr1*^-/-^ (labeled IFNLR^-/-^), and *Ifnar1*^-/-^ (labeled IFNAR^-/-^) mice were infected with 5x10^5^ PFU C2-202 HMPV and euthanized day 1 post-infection. A-J) We quantified total number of lung innate immune cells, including macrophage, monocyte, and dendritic cell subpopulations, in mice described. infection. K-O) We also quantified total numbers of lung epithelial cell populations in mice described. Data shown as mean ± standard deviation. Analyses done by two-way ANOVA. * p<0.05, ** p<0.01, *** p<0.001, **** p<0.0001.

**Figure S11. Optimization of *in vivo* IFN treatment models.** A-B) Optimization of IFN-λ treatment dose. Various doses of IFN-λ were delivered intranasally in 50μL volume. Mice were euthanized day 1 post-treatment, and levels of interferon-stimulated genes MX1 (A) and IRF9 (B) were measured by qPCR of lung homogenates and nasal turbinates. C-D) Optimization of IFN-β treatment dose. Various doses of IFN-β were administered intranasally as described above. qPCR of MX1 (C) and IRF9 (D) expression done for lungs and nasal turbinates of mice collected day 1 post-treatment. Data is normalized to HPRT1 housekeeping gene and the null condition of mock-treated cells by the 2^-∆∆Ct^ method.

**Figure S12. Lung innate populations are altered by IFN-λ or type I IFN treatment.** Mice received mock-, IFN-λ-, or IFN-β prophylaxis 24 hours prior to infection with 5x10^5^ PFU C2-202 and were euthanized day 1-post infection. A-J) We quantified total number of lung innate immune cells, including macrophage, monocyte, and dendritic cell subpopulations, for mice described. K-O) We quantified total number of lung epithelial cell populations in mice described. P) We assessed effects of IFN-λ or type I IFN treatment without HMPV infection (schematic diagram shown). Q) Flow cytometry of lung innate immune and epithelial cell populations altered by IFN treatment alone as described in S9P. Data shown as mean ± standard deviation. Analyses done by one-way ANOVA. * p<0.05, ** p<0.01, *** p<0.001, **** p<0.0001.

**Figure S13. IFN-λ treatment does not contribute to lung inflammatory pathology.** A) Lung histology of HMPV-infected mice that received IFN prophylaxis and were euthanized day 5 post-infection. B) Lung histology of HMPV-infected mice that received IFN treatment and were euthanized day 5 post-infection. Scoring criteria per field included: 0: no inflammation; 1: <25% inflammation; 2: 25-50% inflammation; 3: 50-75% inflammation; 4: >75% inflammation. Score for each sample was added and divided by total number of fields analyzed. C) Representative lung histology images are shown for mice receiving mock-, IFN-λ-, or IFN-β prophylaxis 24 hours prior to 5x10^5^ PFU C2-202 HMPV infection euthanized day 5 post-infection. Analyses done by one-way ANOVA. ****p<0.0001.
