# Supplementary material for: IFN-λ drives distinct lung immune landscape changes and antiviral responses in human metapneumovirus infection
Source: mBio. 2024 Mar 26;15(5):e00550-24. doi: 10.1128/mbio.00550-24 (PMC11077986; doi:10.1128/mbio.00550-24)
Supplement: Table S1 — Antibody table. [file mbio.00550-24-s0004.docx]

**Table S1. Antibody Table**

| Myeloid panel: | | | | | |
| --- | --- | --- | --- | --- | --- |
| Marker | Fluorophore | Species | Clone | Catalog # | Provider |
| CD45 | BV510 | Rat | 30-F11 | 103138 | BioLegend |
| Ly6G | APC-H7 | Rat | 1A8 | 565369 | BD Biosciences |
| CD24 | BUV661 | Rat | MI/69 | 750679 | BD Biosciences |
| MHCII | AF700 | Rat | M5/114.15.2 | 107621 | BioLegend |
| CD64 | BV711 | Mouse | X54-5/7.1 | 139311 | BioLegend |
| CD11c | BUV805 | Hamster | HL3 | 749090 | BD Biosciences |
| CD11b | PE-CF594 | Rat | MI/70 | 562287 | BD Biosciences |
| iNOS | PE-Cy7 | Rat | CXNFT | 25-5920-82 | Thermo-Fisher |
| CD163 | AF532 | Rat | TNKUPJ | 58-1631-80 | Thermo-Fisher |
| Erg2 | PE | Rat | Erongr2 | 12-6691-80 | Thermo-Fisher |
| MAR-1 | Super Bright 600 | Hamster | MAR-1 | 63-5898-82 | Thermo-Fisher |
| CD172a | BUV737 | Rat | P84 | 741819 | BD Biosciences |
| Ly6C | FITC | Rat | AL-21 | 561085 | BD Biosciences |
| Siglec-H | PerCP-eFluor710 | Rat | eBio440c | 46-0333-82 | Thermo-Fisher |
| CD103 | BV785 | Hamster | 2E7 | 121439 | BioLegend |
| XCR1 | BV421 | Mouse | ZET | 148216 | BioLegend |
| Epithelial panel: | | | | | |
| CD31 | FITC | Rat | 390 | 11-0311-82 | Thermo-Fisher |
| CD24 | PerCP-Cy5.5 | Rat | MI/69 | 101823 | BioLegend |
| Podoplanin | PE | Hamster | eBio8.1.1 | 12-5381-82 | Thermo-Fisher |
| EpCAM | PE-Cy7 | Rat | G8.8 | 188216 | BioLegend |
| GS-IB4 | AF594 | Legume | - | I21411 | Thermo-Fisher |
| SSEA-1 | AF405 | Mouse | 480 | sc-21702 | Santa Cruz |
| Pro-SPC | - | Rabbit | - | AB3786 | Sigma-Aldrich |
| Rabbit IgG | AF647 | Goat | - | A-21245 | Thermo-Fisher |
